# *Escherichia coli* EC93 deploys two plasmid-encoded class I contact-dependent growth inhibition systems for antagonistic bacterial interactions

**DOI:** 10.1099/mgen.0.000534

**Published:** 2021-03-01

**Authors:** Marcus Wäneskog, Tiffany Halvorsen, Klara Filek, Feifei Xu, Disa L. Hammarlöf, Christopher S. Hayes, Bruce A. Braaten, David A. Low, Stephen J. Poole, Sanna Koskiniemi

**Affiliations:** ^1^​ Department of Cell and Molecular Biology, Uppsala University, Uppsala, Sweden; ^2^​ Department of Molecular, Cellular and Developmental Biology, University of California Santa Barbara, California, USA; ^†^​ Present address: Department of Biology, University of Zagreb, Zagreb, Croatia; ^‡^​ Present address: Science for Life Laboratory, KTH, Sweden

**Keywords:** competition, contact-dependent growth inhibition, *Escherichia coli*, genome, regulation, toxin, toxic potency

## Abstract

The phenomenon of contact-dependent growth inhibition (CDI) and the genes required for CDI (*cdiBAI*) were identified and isolated in 2005 from an *Escherichia coli* isolate (EC93) from rats. Although the *cdiBAI* ^EC93^ locus has been the focus of extensive research during the past 15 years, little is known about the EC93 isolate from which it originates. Here we sequenced the EC93 genome and find two complete and functional *cdiBAI* loci (including the previously identified *cdi* locus), both carried on a large 127 kb plasmid. These *cdiBAI* systems are differentially expressed in laboratory media, enabling EC93 to outcompete *E. coli* cells lacking cognate *cdiI* immunity genes. The two CDI systems deliver distinct effector peptides that each dissipate the membrane potential of target cells, although the two toxins display different toxic potencies. Despite the differential expression and toxic potencies of these CDI systems, both yielded similar competitive advantages against *E. coli* cells lacking immunity. This can be explained by the fact that the less expressed *cdiBAI* system (*cdiBAI^EC93-2^*) delivers a more potent toxin than the highly expressed *cdiBAI^EC93-1^* system. Moreover, our results indicate that unlike most sequenced CDI^+^ bacterial isolates, the two *cdi* loci of *E. coli* EC93 are located on a plasmid and are expressed in laboratory media.

## Data Summary

The Whole Genome Shotgun project has been deposited at DDBJ/ENA/GenBank under accession PRJNA658456. The GenBank assembly accession is CP061329 for the genome and CP061330 for the pCP127 plasmid. Raw reads from Pacbio sequencing are deposited at the NCBI Sequence Read Archive (SRA) under accession number SRP278798. Ten supplementary figures and seven supplementary tables are available with the online version of this article. The authors confirm all supporting data, code and protocols have been provided within the article or through supplementary data files.

**Impact Statement**

Bacteria live in environments where resources for growth are often limited. To compete for resources, bacteria use multiple mechanisms to inhibit the growth of neighbouring bacteria. One example is contact-dependent growth inhibition (CDI), discovered almost 15 years ago in the *Escherichia coli* isolate EC93. Although CDI has been the focus of many studies over the years, we here present the first comprehensive study of the natural isolate where the phenomenon was first observed. We find that EC93 has not just one but two *cdi* loci, one of which had not been identified before, present on a large plasmid. Bioinformatic analyses of available *E. coli* genome sequences indicate that EC93 is rather unique in having two plasmid-borne *cdi* loci, where both are expressed and active under laboratory conditions. This could potentially explain why CDI was first discovered in this particular strain of *E. coli *. The expression of both *cdi* loci are condition-dependent, probably explaining why the second locus was missed in the original studies. Finally, our analyses show that CdiA toxins with the same toxic activity can differ in toxicity, suggesting that there is interesting molecular biology to be investigated behind the mechanisms of action of the two toxins.

## Introduction

Nutrients are limited in most environments, rendering the capacity to inhibit the growth of rival bacteria a beneficial trait. To increase their relative fitness and chance of survival, most bacteria harbour multiple systems for antagonistic interactions including soluble bacterial toxins like bacteriocins and microcins, as well as contact-dependent growth inhibition (CDI) systems that deliver toxins through the type V and VI secretion systems (T5SS, T6SS) [1–5].

The first CDI system was discovered and characterized in a rat isolate of *Escherichia coli *, designated EC93. Early studies identified three essential genes (*cdiBAI*) that were required for interbacterial growth inhibition. CdiB functions as a transporter that exports the CdiA toxin-delivery protein through the outer membrane of the cell to the cell surface [4, 6, 7]. CdiA forms a β-helical fibre with a receptor-binding domain that specifically recognizes and binds to surface receptors on target bacteria. The C-terminal toxin resides in the periplasm due to secretion arrest [6]. Receptor binding re-starts secretion, facilitating delivery of toxin into target cells. CdiA molecules are classified according to their receptor-binding domains and, so far, three different classes of *E. coli* CdiA proteins have been identified and characterized. CdiA class I molecules bind to BamA, class II to OmpC/OmpF heterotrimers and Class III to Tsx [8–11]. CdiA-CT toxins display diverse activities including ionophores that dissipate the proton motive force (PMF) and nucleases that degrade DNA or RNA [8, 12–17]. To protect themselves from auto-inhibition, CdiI functions as an immunity protein that specifically binds to and blocks the toxicity of its cognate CdiA-CT effector [4, 18].

The mechanism of CDI has been studied extensively using a plasmid-based system expressing the *cdiBAI^EC93-1^* locus, hereafter referred to as *cdi-1*, from its native promoter on a medium-copy vector in laboratory *E. coli* strains [4, 6, 9–11]. Here we analyse the original EC93 isolate, measuring CDI expression in the native context. Notably, we find that the genome of EC93 contains two intact and fully functional *cdiBAI* loci. Both *cdiBAI* loci are located on a large 127 kb plasmid, inhibiting the growth of *E. coli* strains lacking cognate *cdiI*-encoded immunity. Moreover, the two *cdiBAI* loci have an ≈4.5-fold difference in expression but achieve the same relative growth inhibition, which we demonstrate is probably due to differences in the toxicity of their CdiA-CT effectors.

## Methods

### Strains and growth conditions

The bacterial strains, plasmid constructs and oligos used in this study are listed in Tables S1–S3 (available in the online version of this article). Strains were grown at 37 °C and with shaking at 200 r.p.m. in M9 rich media: 1× M9 salts supplemented with 0.4 % glucose, 0.2 % cas-amino acids, 2 mM MgSO_4_, 0.1 mM CaCl_2_ and 50 µM FeCl_3_; or in Lysogeny broth (LB): 10 g l^−1^ tryptone, 5 g l^−1^ yeast extract and 10 g l^−1^ NaCl. Agar was added at 15 g l^−1^ for solid media plates. Media were supplemented with antibiotics when applicable as follows: ampicillin 100 mg l^−1^, chloramphenicol 18 mg l^−1^, kanamycin 50 mg l^−1^, streptomycin 100 mg l^−1^, spectinomycin 50 mg l^−1^ and tetracycline 25 mg l^−1^.

### Whole genome sequencing, assembly and annotation

Chromosomal DNA from *E. coli* strain EC93 was isolated using the Qiagen midi-prep (Genomic-tip 100/G) according to the manufacturer and whole genome sequencing was performed at the SciLife laboratory, Uppsala, Sweden. PacBio libraries were produced using the SMRTbell Template Prep Kit 1.0 according to the manufacturer’s instructions (Pacific Biosciences). Libraries were subjected to exo treatment and PB AMPure bead wash procedures for clean-up before size selection with the BluePippin system (Sage Sciences) with a cut-off value of 6500 bp. The libraries were sequenced on the PacBio RS II instrument using C4 chemistry, P6 polymerase and 240 min movie time in one SMRTcell.

The Pacbio reads were assembled using HGAP3 from SMRTportal (PacBio) with default settings, producing two circular contigs at size of 4 825 910 and 127 456 bp. The larger contig was determined to be the genomic contig, and the sequence was then re-oriented to start at 100 bp upstream of dnaA.

The genome was annotated using the NCBI Prokaryotic Genome Annotation Pipeline (PGAP) (2020-09-04) [19]. The *cdi* regions of interest were manually curated. This Whole Genome Shotgun project has been deposited at DDBJ/ENA/GenBank under accession PRJNA658456, and the GenBank assembly accession is CP061329 for the genome and CP061330 for the pCP127 plasmid. Raw reads from PacBio sequencing have been deposited at the NCBI Sequence Read Archive (SRA) under accession number SRP278798.

### Estimation of genomic locations of *cdi* systems

The EC93 *cdiA1* nucleotide sequence was used to query against the NT database (2020-10-07) using ‘blastn -task blastn’ to find all the sequences that have similarity to the *cdiA* gene. Fragmented and non-specific blastn hits were removed [(alignment length <2000 and percentage identity <80.5) or (alignment length <3000 and *e*-value=0) or (alignment length <1000)], leaving 608 hits. Their corresponding 272 genomic sequences were downloaded from NCBI. Filtered blastn matches were then parsed and the sequences of the *cdiA* region with 20 kbp upstream as well as 30 kbp downstream were extracted from the downloaded sequences. The selected sequences were then annotated with prokka v.1.14.6 [19], using EC93 annotation as a reference to get a proper annotation of the *cdi* region. If the sequence description contains ‘complete genome’ or ‘chromosome’ or has a size greater than 4 Mbp, then the sequence is determined to be of chromosomal origin, and if the sequence description contains ‘plasmid’, then the sequence is determined to be plasmid-encoded. Only the sequences with intact *cdiAs* (size >9070 bp) were considered in this analysis. In addition, the full genomes in the database are not a random representation of bacterial genomes. If there are many duplicated or identical strains this could skew the data in either direction. Thus, the annotations were parsed for generation of unique, non-duplicated strain sequences (Tables 1, 2 and S7).

**Table 1.** The genome location of *E. coli cdi* systems The genomic locations of 190 *E. coli cdi* systems, determined by blastn search using the *cdiA1* gene from EC93 as query.

Location in the genome	No. of CDI systems (% of total intact *E. coli* CDI systems)
Chromosome	177 (93.2 %)
Plasmid	13 (6.8 %)

**Table 2.** Plasmids identified to contain intact *E. coli cdi* systems List of all 12 *E. coli* plasmids identified to contain at least one *cdi* system by blastn search, using the *cdiA1* gene from EC93 as query.

Identification number	Number of identified CDI systems	Plasmid size (bp)	Origin of plasmid (* E. coli* strain)
CP027343.1	1	131 410	2014C-4587
CP010214.1	1	146 496	M15
CP027453.1	1	159 611	2014C-3338
CP009107.1	1	161 447	94-3024
CP023542.1	1	161 452	CFSAN002236
CP011019.1	1	207 265	CI5
CP010181.1	1	201 930	M1
CP010207.1	1	138 950	M11
CP010184.1	1	200 925	M3
CP023164.1	1	122 641	RM10809-3
CP010192.1	1	162 720	M8
CP061330.1	2	127 456	EC93

### Construction of plasmids and chromosomal constructs

All constructs were verified by PCR and sequencing. Detailed information of the different constructs is presented in the supplementary material (Supplementary data).

### Competition assay

Inhibitor and target cells were individually grown overnight in LB before being mixed at a ratio of 10 : 1 (if not stated otherwise) and either diluted 1 : 100 in liquid media or 20 µl was spotted on solid media. For liquid media competitions, cells were co-cultured for 16 h (if not stated otherwise) at 37 °C with 200 r.p.m. shaking. The c.f.u. ml^-1^ values of inhibitor and target cells at 0 and 16 h were enumerated by serial dilutions followed by plating on LB solid media containing appropriate antibiotics. For competitions on solid media, cells were co-cultured for 16 h at 37 °C before the entire colony was suspended in 1× PBS pH 7.4, and inhibitor and target cells were enumerated as above. Competitive indices were calculated as the ratio of inhibitor to target cells at the end of the co-culture (16 h) divided by the ratio at the beginning of the co-culture (0 h). The competitive indices for at least three independent experiments are reported ±sem (if not stated otherwise). Statistical significance was calculated using the unpaired, two-tailed, Student's *t*-test.

### CdiA-CT toxic effect assay

We mixed red fluorescent protein-labelled (RFP) MG1655 target cells with inhibitor cells expressing *E. coli* EC93 *cdi-1* locus constructs modified to deliver either the EC93 CdiA-CT1 toxin (SK2968), the EC93 CdiA-CT2 toxin (SK2974) or the previously characterized NC101 tRNase toxin (SK2971) [20]. Strains were individually grown overnight in LB before diluting 1 : 1000 in M9Glu+casAA. Inhibitor and target cells were grown to log-phase (OD_600_ of 0.3–0.4) and mixed at a ratio of 5 : 1 before being co-cultured for 1 h at 37 °C with shaking (200 r.p.m.). A final concentration of 50 µM DiBAC_4_(3) was added and cells were incubated for an additional 30 min. Samples were then diluted 1:200 in 1× PBS (pH 7.4), vigorously mixed, and analysed by a MACSQuant VYB flow cytometer, using filter B1 (525/50 nm) (Miltenyi Biotec). Flow rate was set to allow for ≈2000 events per second and at least 20 000 target cell (RFP+) events were collected. The fraction of DiBAC_4_-stained target cells (RFP+) were analysed by FlowJo Software (FlowJo) for each population (*n*=4–6).

### Flow cytometry assay of *cdi* transcriptional reporters

EC93 *Δcdi-1* with *cdiB2-sYFP2-cdiA2* or EC93 *Δcdi-2* with *cdiB1-sYFP2-cdiA1* reporter constructs were diluted 1:400 into M9Glu+casAA from an LB overnight culture and grown to log phase (OD_600_ of 0.2–0.3). Samples were then diluted 1:200 in 1× PBS and analysed by a MACSQuant VYB flow cytometer, using filter B1 (525/50 nm) (Miltenyi Biotec). Flow rate was set to allow for ≈2000 events per second and at least 100 000 events were collected. Background fluorescence was measured using EC93 *Δcdi-1 Δcdi-2* cells and subsequently subtracted from all sYFP2 cell measurements. The median, single-cell sYFP2 intensity was calculated by FlowJo Software for each EC93 population and later used to calculate the relative *cdi* gene expression.

### CdiA cell-surface immunofluorescence and flow cytometry

EC93 *Δcdi-1*, EC93 *Δcdi-2* or EC93 *Δcdi-1 Δcdi-2* constructs were diluted 1:400 into M9Glu+casAA from an LB overnight culture and grown to log phase (OD_600_ of 0.2–0.3). Formaldehyde was added (1.5 % final concentration), and samples were incubated at 23 °C for an additional 15 min to cross-link CdiA molecules at the cell surface to CdiB in the outer membrane. Glycine was added (500 mM final concentration) and the samples were incubated for an additional 15 min to stop the cross-linking reaction. Cells were pelleted at 3000 ***g*** for 10 min and washed once with PBS pH 7.4, followed by another pelleting at 3000 ***g*** for 10 min. The cell pellets were then re-suspended in PBS pH 7.4 containing polyclonal anti-CdiA antibodies (1:100 dilution) [16]. Cells were incubated for 1 h with shaking at 200 r.p.m., then pelleted at 3000 ***g*** for 10 min and re-suspended in PBS containing goat anti-rabbit IgG conjugated with Alexa Fluor 594 (Abcam; ab150080) at 1:200 dilution. After an additional 1 h of incubation, with shaking, cells were again pelleted at 3000 ***g*** for 10 min and re-suspended in PBS pH 7.4, diluted 1:200 and then mixed and analysed by a MACSQuant VYB flow cytometer, using Y2 filters (615/20 nm) (Miltenyi Biotec). Flow rate was set to allow for ≈2000 events per second and at least 100 000 events were collected. Background fluorescence and CdiA non-specific antibody binding was measured using EC93 *Δcdi-1 Δcdi-2* cells, which were used to normalize measurements taken from EC93 *Δcdi-1* and EC93 *Δcdi-2* cells. The median, single-cell Alexa Fluor 594 intensity was calculated by FlowJo Software (FlowJo) for each EC93 population and later used to calculate relative CdiA cell-surface expression.

## Results

### The EC93 genomic sequence reveals two CDI systems located on a large 127 kb plasmid

**Fig. 1.**
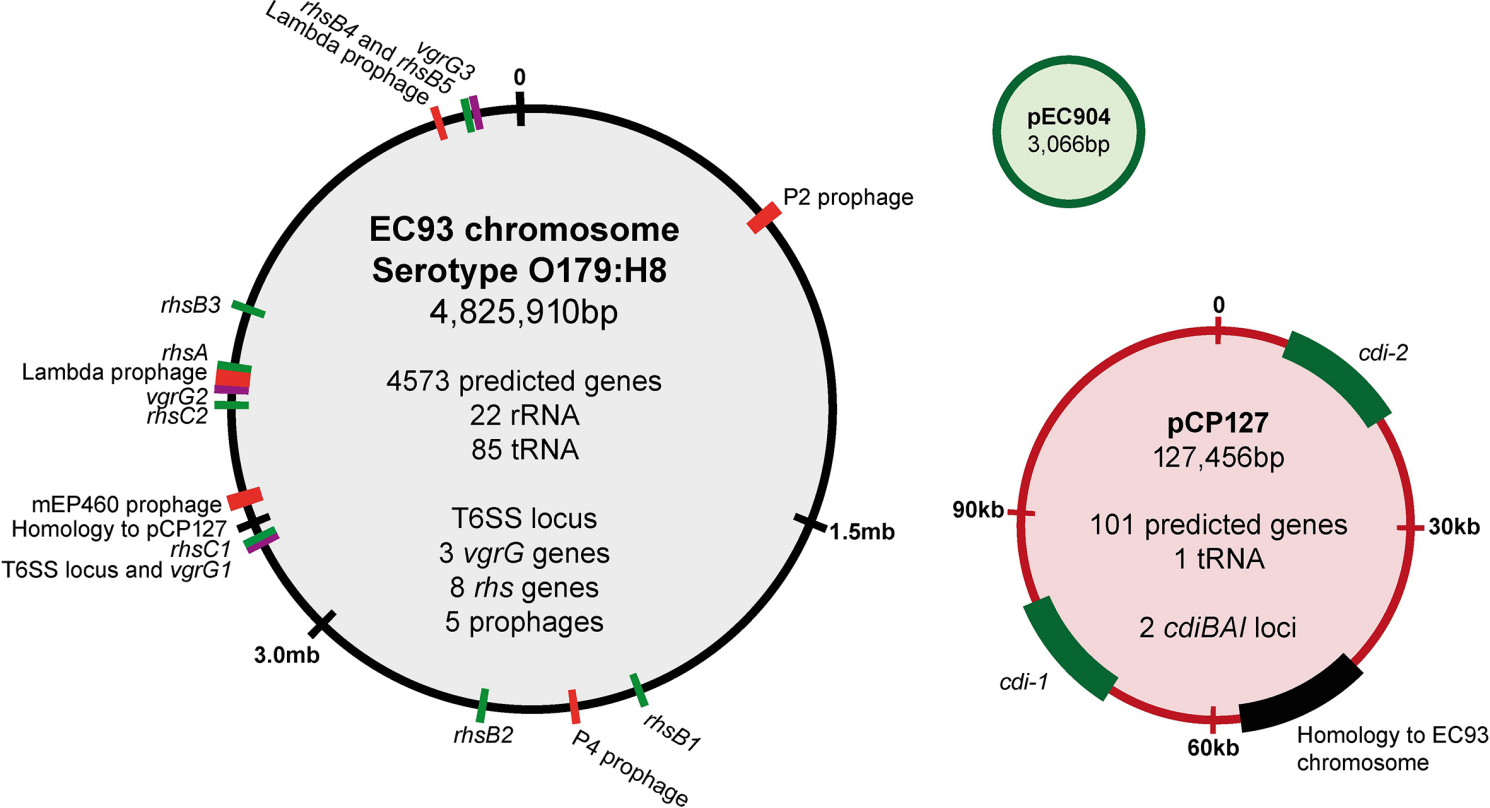
The EC93 genome. Schematic representation of the chromosome and two plasmids (pEC904 and pCP127) of EC93 with *cdi* and *rhs* genes shown in green, *vgrG* genes shown in purple and prophages shown in red. The relative size of plasmids and chromosome illustrations are not to scale.

The phenomenon of bacterial CDI was discovered 15 years ago in an *E. coli* strain isolated from rats, designated as EC93 [4]. To gain further insight into CDI mediated by EC93, we carried out PacBio genomic sequencing, assembling the genome into three contigs: a 4.8 Mb chromosome; a large 127 kb plasmid with an IncFIB-like origin, not previously known (NCBI), which we designated pCP127 (CDI Plasmid 127 kb); and a small, previously known, 3 kb plasmid, pEC904 [21] (Fig. 1). Besides the previously identified CDI system, designated as *cdi-1* here, we also identified an additional CDI system, *cdiBAI^EC93-2^*, hereafter referred to as *cdi-2*. Both CDI systems are located on the pCP127 plasmid. This plasmid contains an approximately 47 kb region with sequence homology to a previously described plasmid present in an *E. coli* isolate originating from a faecal sample (CP010207), and additional regions including those with homology to K88 fimbrial and RTX toxin genes (Table S4). Additional chromosomally located genes that could function in growth competition were identified including eight *rhs* rearrangement hotspot loci and a T6SS locus (Fig. 1). Plasmid pCP127 also contains additional fitness factors, with their functions listed in Table S4. One such factor, the multidrug efflux pump MfdA, was previously shown to be active against some of the antibiotics used in the current study [22]. However, the measured minimum inhibitory concentration (MIC) of EC93 was below the experimental concentrations for all antibiotics used here (Table S5). We could also observe five prophages that appeared to be intact (Fig. 1). Since prophages can contribute to growth inhibition of closely related strains lacking the phage, we investigated the stability of these prophages by subjecting EC93 to UV light of varying intensity (Table S6). No release of phage could be detected through plaque assays on MG1655, demonstrating that either all prophages in EC93 are stable when exposed to DNA damage, or that none of the prophages can infect MG1655.

A comparative analysis of the CdiB, CdiA and CdiI coding sequences of *cdi-1* and *cdi-2* showed that they are distinct (Figs S1–S3). The two CdiB proteins are similar in sequence, with some heterogeneity in their N-termini (Fig. S1). Both CdiA proteins are class I molecules, based on identical receptor-binding domain regions (red-box), but differ in their N-terminal regions, which are predicted to encode filamentous haemagglutinin repeats (FHA-1) (Fig. S2). These domains are known to be important structural domains that vary in both sequence and length between CdiA molecules [6]. In addition, the two CdiA proteins show sequence heterogeneity in the region downstream of the receptor-binding domain (red-box), a region known to be variable between CdiA [6] (Fig. S2). Finally, the CdiA-CT toxin domains, which in *E. coli* are demarcated by the conserved VENN motif, and the corresponding CdiI immunity proteins share little to no homology (Figs S2 and S3), suggesting that the CdiA1 and CdiA2 toxins may have distinct biological activities.

To estimate the frequency that *cdi* systems are found on bacterial chromosomes versus plasmids, we performed a tblastn search of all bacterial genomes, using the EC93 *cdiA1* nucleotide sequence as a query to find all sequences that have similarity to the *cdiA* gene. Our analyses showed that the majority of *cdi* systems in *E. coli* are chromosomally located (Tables 1 and S7).) Out of 190 unique, non-duplicated strain sequences, of *E. coli cdi* systems only 13 (6.8 %) were located on a plasmid. Of these *cdi*-containing plasmids only the EC93 pCP127 plasmid contained two separate *cdi* loci (Table 2), demonstrating that the pCP127 plasmid is rare even compared to other *cdi*-containing plasmids.

### The EC93 *cdi-*2 system is functional

**Fig. 2.**
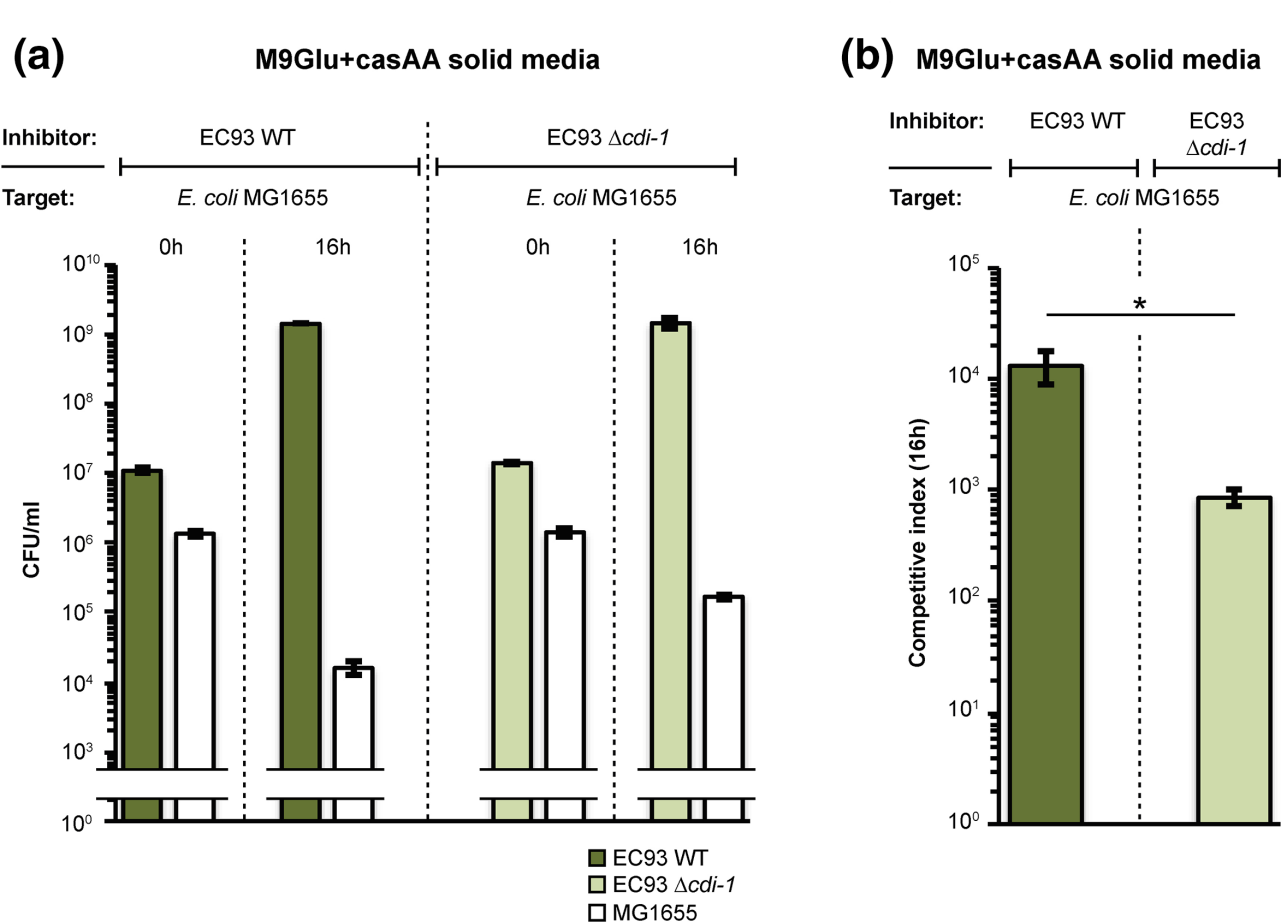
EC93 lacking *cdi-1* still outcompetes *E. coli* MG1655. (**a**) Average c.f.u. ml^-1^ of inhibitor (EC93) and target (MG1655) cells at 0 and 16 h following mixing on M9Glu+casAA solid medium (*n*=3 biological replicates ±sem). (**b**) Average competitive index calculated from the data in Fig. 2a. Statistical significance was determined using a two-tailed, unpaired *t*-test (*P<0.05).

As a first step in determining if the *cdi-2* system is functional in EC93, we examined CDI activity when bacteria were co-cultured on M9 glucose solid medium supplemented with casamino acids (casAA) [10]. EC93 outcompeted the laboratory *E. coli* strain MG1655 on this medium by over 4-log based on the competitive index (CI) (Fig. 2a, b). This result is consistent with previous results showing that EC93 expresses a functional *cdiBAI* system (*cdi-1*) that is active against laboratory *E. coli* strains [23, 24]. However, EC93 cells lacking *cdi-1* still outcompeted *E. coli* MG1655 with over 3-log of growth inhibition (Fig. 2a, b). This is in contrast to previous findings, where an EC93 *cdi-1* mutant showed almost complete loss of growth inhibitory activity when cells were co-cultured on LB solid medium [23, 24]. These results suggest that EC93 possesses an additional growth inhibitory system(s) that is active on M9Glu+casAA solid medium. To relate our experiment to previous findings, we repeated previously described co-cultivation experiments on LB solid media using the same competition assay and target strains as used previously [23]. Because this competition assay is somewhat different from that used throughout the present study, the CIs are not directly comparable. EC93 cells outcompeted *E. coli* X90 with ~3- log growth inhibition on both LB and M9Glu+casAA solid media (Fig. S4). As expected from previous findings, EC93 cells lacking *cdi-1* were not able to outcompete X90 cells on LB solid medium. In contrast, EC93 *cdi-1* mutants outcompeted X90 cells to the same extent as wild type EC93 cells on M9Glu+casAA medium. Together, these results show that EC93 deploys a growth inhibition system in addition to *cdi-1* that is functional on M9Glu+casAA solid medium.

**Fig. 3.**
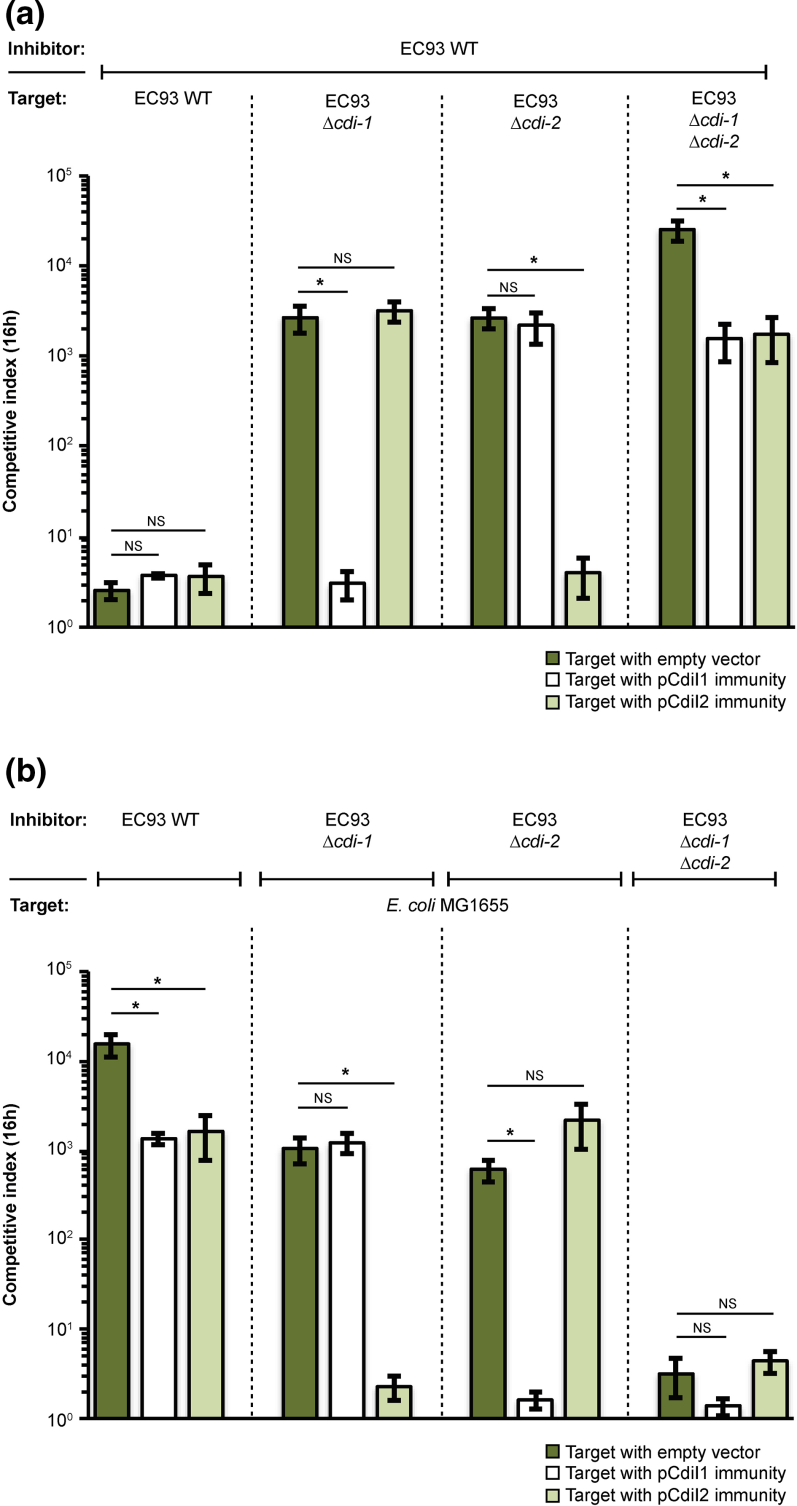
EC93 expresses two functional CDI systems on M9Glu+casAA solid medium. (**a**) Average CIs of inhibitor (EC93) and target (EC93 *cdi-1* and *cdi-2* mutants) cells after 16 h of co-culture on M9Glu+casAA solid medium (*n*=3 biological replicates). Target cells were transformed with pCDF empty vector or pCDF vector expressing either CdiI1 or CdiI2. (**b**) Same co-culturing experiment as in Fig. 3a but with MG1655 as target and EC93 or EC93 *cdi* mutants as inhibitors (*n*=3 biological replicates ±sem). Statistical significance was determined by two-tailed, unpaired *t*-test (*P<0.05).

The results above suggested the possibility that the *cdi-2* locus in EC93 is active. Therefore, we carried out growth competitions between EC93 and EC93 mutants lacking one or both of the *cdi* loci. When cells were co-cultured on M9Glu+casAA solid media, EC93 outcompeted EC93 *cdi* mutants 1000 to 10 000-fold (Fig. 3a). As expected, expression of cognate, but not non-cognate immunity protein protected cells from growth inhibition (Fig. 3a). Growth inhibition of the EC93 mutant lacking both *cdi* loci could not be rescued by the expression of either CdiI immunity proteins alone, further demonstrating that both *cdi* systems can mediate growth inhibition under these conditions (Fig. 3a).

To determine if the ability of EC93 to outcompete *E. coli* MG1655 on M9Glu+casAA solid media (Fig. 2) was mediated by *cdi-2*, we competed EC93 and EC93 mutants lacking one or both *cdi* loci against MG1655 target cells (Fig. 3b). To verify that the observed growth inhibition was a result of CdiA toxin delivery, we expressed the cognate immunity protein of each CdiA-CT toxin in MG1655. Neither *cdiI* immunity alone was able to fully rescue the observed growth inhibition when MG1655 was competed against WT EC93 (Fig. 3b). In contrast, either immunity protein alone was able to fully rescue the observed growth inhibition when MG1655 was competed against EC93 strain(s) expressing only the cognate CdiA protein (Fig. 3b). Moreover, EC93 cells lacking both *cdi* loci did not outcompete MG1655 cells, suggesting that *cdi-*1 and *cdi-*2 are the only growth competition systems active under the conditions analysed here (Fig. 3b). Together, these results show that both *cdi* loci of EC93 are fully functional under similar growth conditions and that EC93 inhibitor cells armed with both *cdi* systems outcompete target strains about 10-fold more than EC93 armed with only one *cdi* system (Fig. 3a, b). Thus, both *cdi* systems contribute to the growth inhibition capability of EC93.

### Comparative analysis of the *cdi-1* and *cdi-2* growth inhibition systems

Previous work showed that CdiA1, encoded by *cdi-1*, contains a receptor-binding domain (RBD) that interacts with the essential outer-membrane protein BamA to facilitate effector delivery [11, 25]. The 100 % protein identity between the CdiA RBD of CdiA2 (encoded by *cdi-*2) with the CdiA1 RBD (Fig. S2) indicates that CdiA2 probably also uses BamA as a receptor. To test this hypothesis, we carried out growth competitions using *E. coli* target cells expressing native BamA (BamA^MG1655^) or a previously described non-cognate BamA receptor from *Salmonella enterica* serovar Typhimurium (BamA^Sty^) [26]. Previous work showed that target cells expressing BamA^Sty^ are resistant to growth inhibition caused by CdiA1 due to altered extracellular loop residues important for binding [23]. Our results showed that growth inhibition mediated by CdiA2 (see *E. coli *Δ*cdi-1*) was fully dependent on BamA^MG1655^, as was growth inhibition by CdiA1 (Fig. S5). *E. coli* EC93 lacking both *cdi* systems did not inhibit targets, showing that under these conditions the two *cdi* systems account for all growth inhibition observed.

### CdiA-CT2 dissipates the PMF of target bacteria

**Fig. 4.**
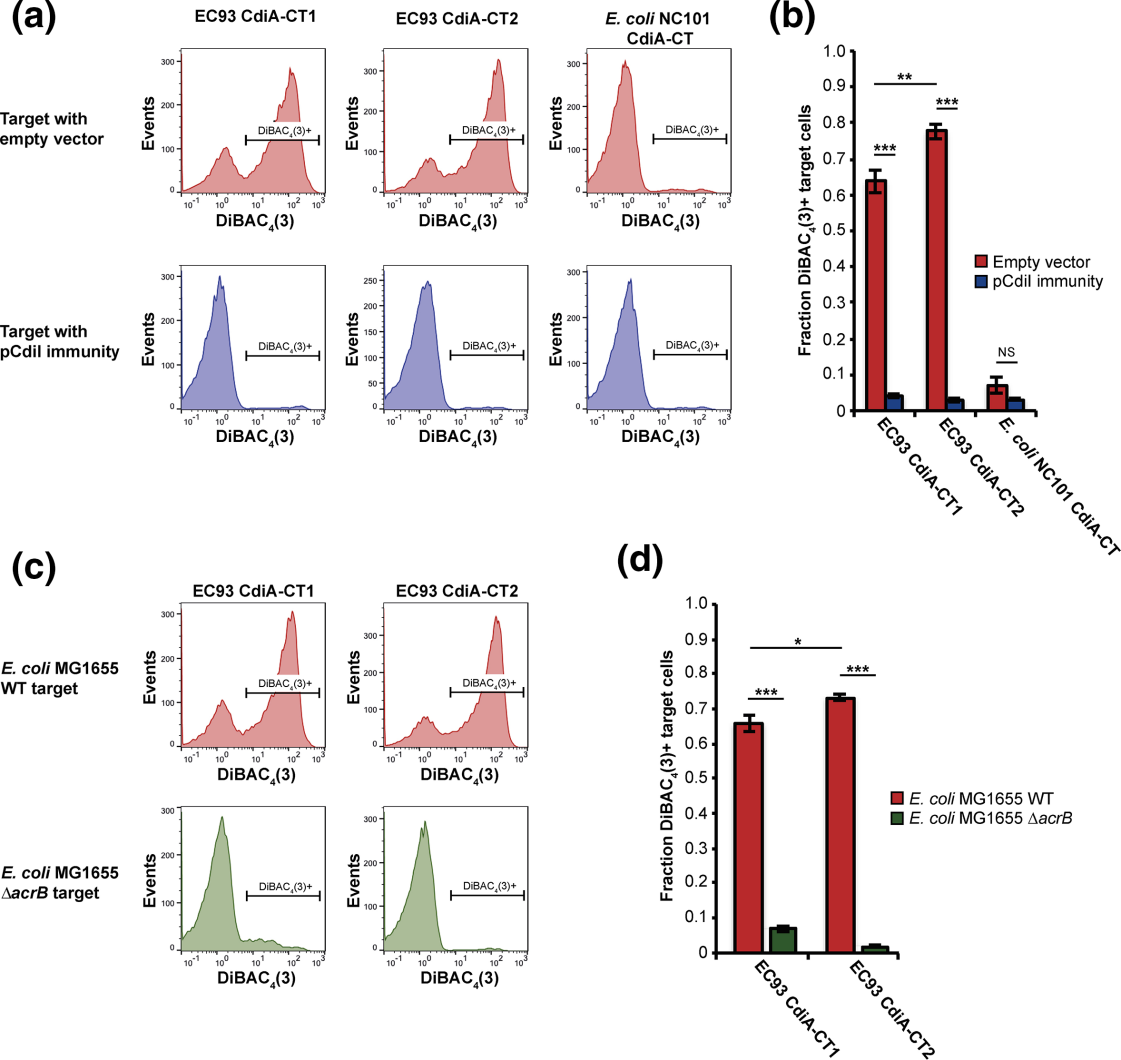
The CdiA-CT1 and CdiA-CT2 toxins are membrane ionophores that require AcrB for activity. Membrane depolarization analyses using DiBAC_4_(3) (see Materials and Methods). *E. coli* MG1655 inhibitor cells expressing the CdiA-CT1, CdiA-CT2 or CdiA-CT^NC101^ toxins (see Materials and Methods), depicted at the top of the panels, were mixed with *E. coli* MG1655 target cells in M9Glu+casAA liquid medium, then analysed by flow cytometry. In the lower three panels, targets expressing cognate CdiI immunity proteins were analysed. (**b**) Quantification of the data in (a) as the fraction of target cells that were depolarized (*n*=6 biological replicates ±sem). (**c**) AcrB-dependence of membrane depolarization. Analysis was carried out as in (a) using *E. coli* delivering the EC93 CdiA-CT1 or CdiA-CT2 toxins (top of panels) and wild-type or *ΔacrB* target cells (left of panels). (**d**) Quantification of the data in (c) as the fraction of target cells that were depolarized (*n*=4 biological replicates ±sem). Statistical significance was determined by two-tailed, unpaired *t*-test (*P<0.05, **P<0.005, ***P<0.0005).

The EC93 CdiA1 carboxy-terminal domain (CdiA-CT1) was previously identified as an ionophore toxin, dissipating the PMF of susceptible targeted bacteria [27, 28]. To identify the toxic activity of CdiA-CT2, we constructed a mariner transposon pool in *E. coli* MG1655 and enriched for mutants resistant to CdiA-CT2 by repeatedly competing the transposon pool with *E. coli* EPI100 inhibitor cells delivering the CdiA-CT2 toxin via BamA receptor using a CdiA1-CT2 chimera. After three rounds of enrichment, we isolated CDI-resistant mutants and used semi-random arbitrary PCR to identify the insertion sites of the transposons providing resistance towards the CdiA-CT2 toxin. We identified six transposon insertions in five distinct sites within the *acrB* gene (Fig. S6a), coding for an inner membrane multidrug efflux pump. To verify that CdiA-CT2 requires AcrB to mediate toxicity, we competed *ΔacrB* mutants of *E. coli* EPI100, transformed with an AcrB expression vector or an empty vector control, against inhibitor cells expressing CdiA-CT2. Cells expressing CdiA-CT2 outcompeted EPI100 target cells by 3-log on LB solid media (Fig. S6b), but did not inhibit the growth of AcrB-deficient *E. coli* cells (Fig. S6b), confirming the AcrB dependence of the CdiA-CT2 toxin. Because the CdiA-CT1 toxin was shown previously to require AcrB to dissipate the PMF [8], we determined if CdiA-CT2 also displays ionophore activity. *E. coli* expressing chimeric CdiAs [13] delivering either CdiA-CT1, CdiA-CT2 or the previously characterized tRNase toxin of *E. coli* NC101 were co-cultured with fluorescently labelled target cells, followed by addition of the fluorescent dye DiBAC_4_(3). DiBAC_4_(3) is selectively taken up by bacterial cells in which the PMF has been dissipated [29, 30]. Flow cytometry analysis showed that 63 and 77 % of target cells co-cultured with CdiA-CT1- or CdiA-CT2-expressing inhibitor cells respectively, became stained with DiBAC_4_(3). DiBAC_4_(3) uptake was blocked by expression of cognate CdiI, showing its direct dependence on *cdi* toxin activity. In contrast, less than 7.5 % of targets co-cultured with inhibitor cells expressing the tRNase toxin were stained (Fig. 4a, b). Furthermore, target cells lacking *acrB* were not stained by DiBAC_4_(3) when co-cultured with inhibitor cells expressing either toxin (Fig. 4c, d). Moreover, the fraction of target cells with a depolarized membrane was consistently higher (7–13 %) when cells were intoxicated by the CdiA-CT2, compared to the CdiA-CT1 (Fig. 4a–d). Together, these results indicate that CdiA-CT2, like CdiA-CT1, is an ionophore that requires the inner membrane protein AcrB for activity.

### CdiA1 and CdiA2 are differentially expressed and differ in potency

**Fig. 5.**
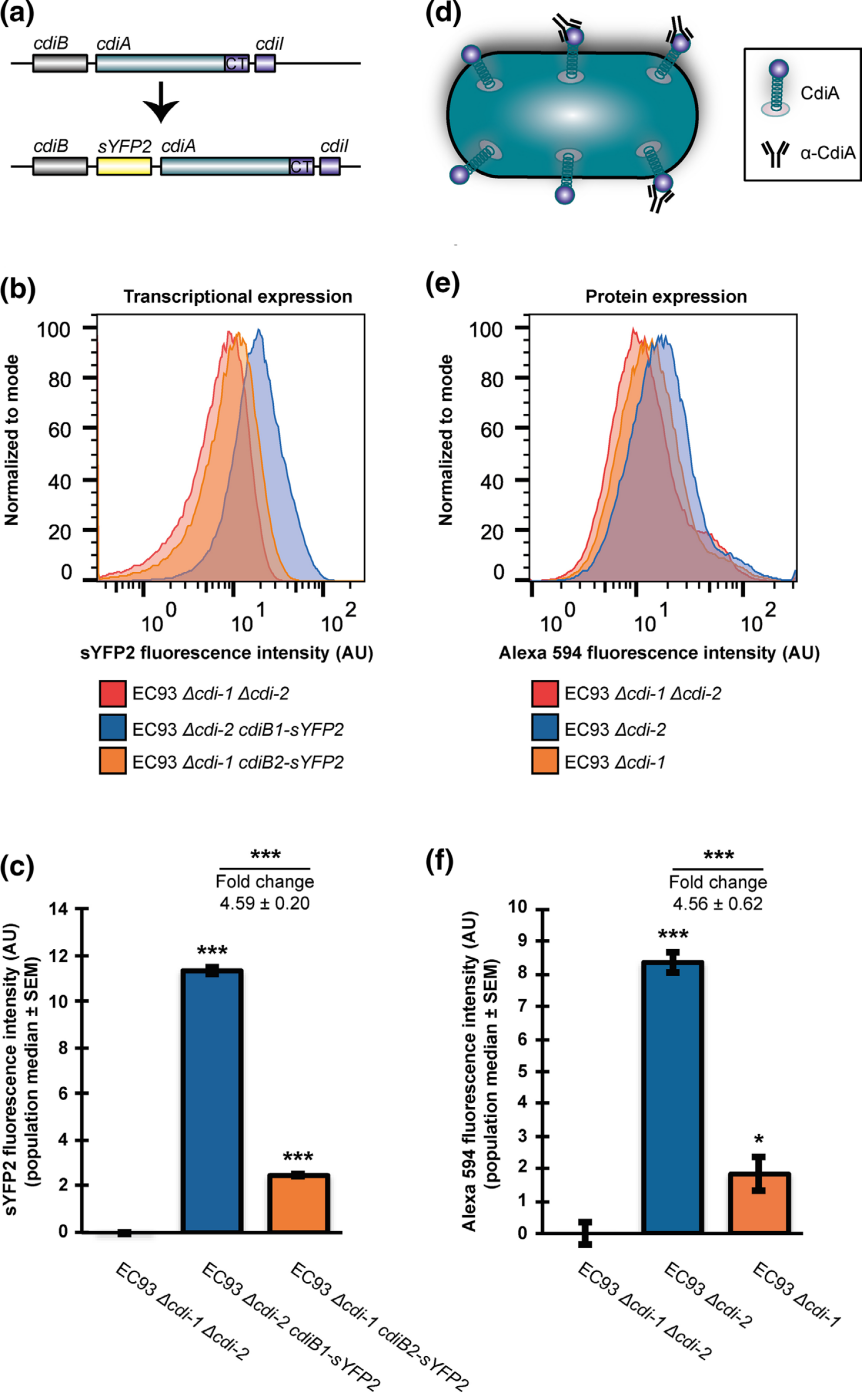
The *cdi1* and *cdi2* systems of EC93 are differentially expressed. (**a**) Schematic representation of the transcriptional reporter constructs used to measure *cdi* transcriptional activity. (**b**) Relative transcriptional activity of the *cdi-1* and *cdi-2* loci in EC93 determined by sYFP2 fluorescence (see Materials and Methods). (**c**) Quantification of the data presented in (b) (*n*=3 biological replicates ±sem). (d) Schematic representation of the immunofluorescence (IF) approach used to measure CdiA abundance on the surface of EC93. (**e**) Relative CdiA surface abundance on EC93 cells (see Materials and Methods). (**f**) Quantification of the data presented in (e) (*n*=3 biological replicates ±sem). Statistical significance was determined by two-tailed, unpaired *t*-test (*P<0.05, **P<0.005, ***P<0.0005).

While both CDI systems of EC93 show similar growth inhibition phenotypes on solid media and deliver toxins with similar functions, the promoter regions of *cdi-1* and *cdi-2* differ in sequence (Fig. S7a). To investigate if this difference in promoter sequences also reflected a difference in transcription factor binding sites, we bioinformatically analysed the putative promoter regions of the *cdi-1* and *cdi-2* loci using the bacterial sigma70 promoter recognition program BPROM [31] (Fig. S7b, c). From this analysis we could observe that both promoters contained several putative transcription factor binding sites and these suggested transcription factors were different for the two loci (Figs. S7b, c), indicating that the two *cdi* loci of EC93 probably have different transcriptional regulation. To experimentally measure the transcriptional expression of each *cdi* locus, a fluorescent marker (sYFP2) was inserted between *cdiB* and *cdiA*, constructing transcriptional reporters in EC93 *Δcdi* mutants lacking the heterologous *cdi* locus (Fig. 5a). Analysis of fluorescence intensity by flow cytometry indicated that the level of transcription of *cdi-1* is about 4.5-fold higher than that of *cdi-2* on M9Glu+casAA medium (Fig. 5b, c). To determine if the increased transcription observed for *cdi-1* is reflected in an increased CdiA expression level, we measured CdiA expression by quantitative cell-surface immunofluorescence (IF), using α-CdiA antibodies that bind to the conserved central region of both CdiA molecules (Fig. 5d). The relative, single-cell CdiA expression of EC93 cells lacking one of the two *cdi* loci was measured by flow cytometry post-antibody labelling (Fig. 5e). Based on this analysis, CdiA1 was expressed about 4.5-fold higher than CdiA2 (Fig. 5f), closely paralleling the transcription data (Fig. 5c). To further verify that the two CdiA proteins are differentially expressed, we analysed CdiA expression from whole-cell lysates of EC93 WT cells and mutants lacking either, or both, *cdi* loci, by SDS-PAGE. Similar to results shown in Fig. 5, we observed a 3.5-fold higher expression of CdiA1 compared to CdiA2 (Fig. S8). In addition, the combined expression of CdiA in EC93 Δ*cdiA1* (CdiA2 only) and Δ*cdiA2* (CdiA1 only) almost matched (91 % of that in WT) the total expression of CdiA in EC93 WT, suggesting that inactivation of either locus does not significantly affect CdiA expression.

To determine if this differential *cdi* expression is dependent upon the growth medium, we measured *cdi* transcription in LB broth, using the same reporter constructs shown in Fig. 5a. Both *cdi* loci were expressed about 1.5- to 3-fold lower in LB compared to M9Glu+casAA (Fig. S9a, b). While *cdi-2* expression was statistically significant compared to the background fluorescence of the negative control (EC93 *ΔcdiA1,ΔcdiA2*), it is possible that the very low *cdi-2* expression we observe in LB media is below the threshold for what is required to mediate growth inhibition. This could explain why we, and others, have not previously observed a *cdi-2*-mediated growth inhibition on LB solid medium (Fig. S4).

**Fig. 6.**
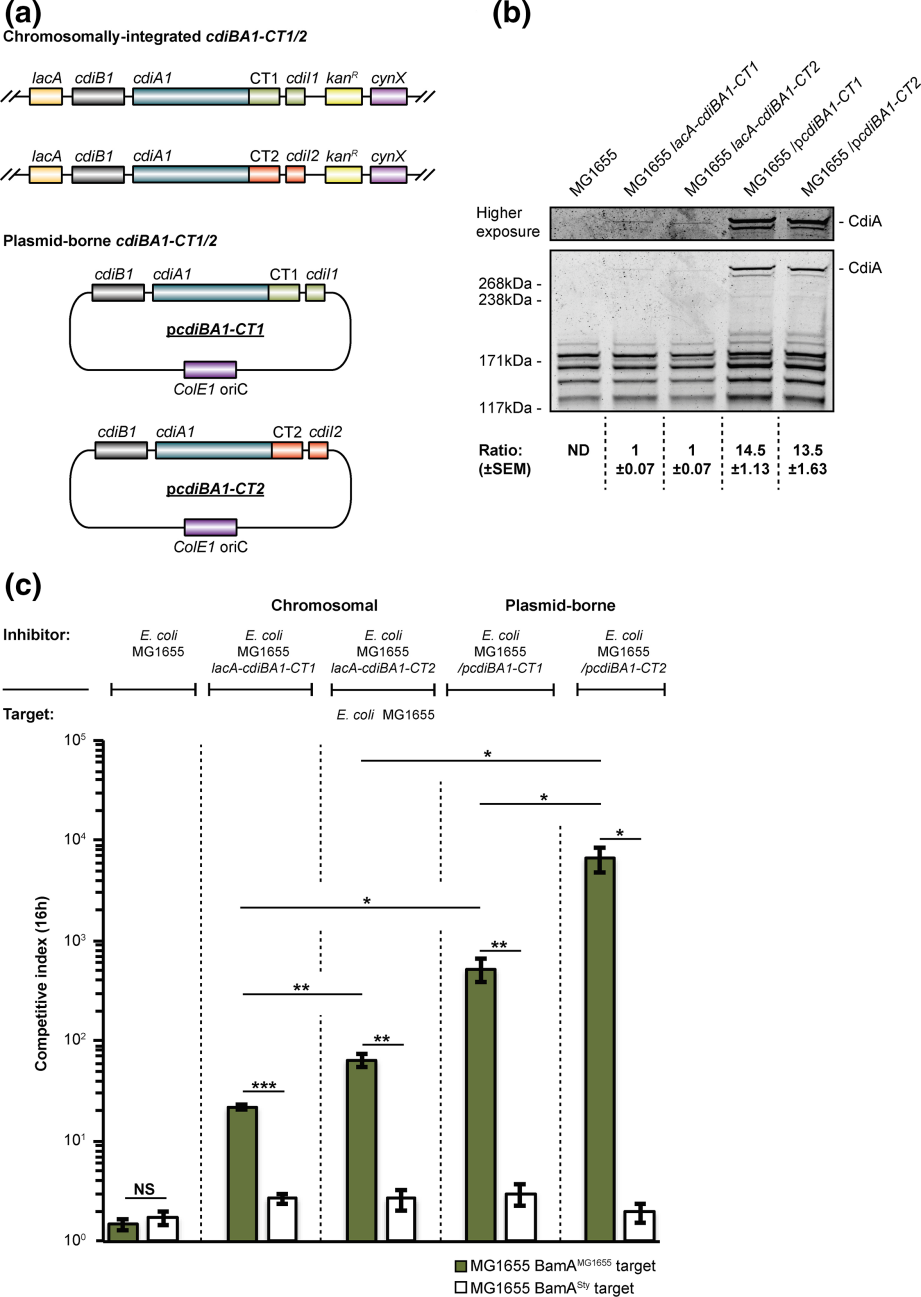
CdiA expression levels correlate with growth inhibition phenotypes for both CdiA-CT1 and CdiA-CT2 toxins. (**a**) Schematic representation of the two chromosomally integrated *cdi* loci and the two expression plasmids used to deliver the CdiA-CT1 or CdiA-CT2 toxins to target cells. (**b**) Expression of CdiA (≈319 kDa) in *E. coli* MG1655 (*cdi* ^-^), MG1655 with chromosomally integrated *cdi-1* or a *cdiA1-CT2* chimaera (*lacA-cdiBA1-CT1/2*), and MG1655 expressing *cdi-1 or a cdiA1-CT2* chimaera from a medium-copy plasmid (p*cdiBA1-CT1/2*) were analysed by SDS-PAGE. Total protein was visualized by SYPRO Ruby staining and relative protein abundance was calculated by densitometry using ImageJ (*n*=4 biological replicates ±sem). (**c**) Average CIs of the inhibitor cells, with either chromosomal or plasmid-borne *cdiBA1-CT1/2* loci, shown at the top with *E. coli* MG1655 target cells with cognate BamA^MG1655^, or non-cognate BamA^Sty^ CdiA receptor (bottom key). Inhibitor cells and target cells were co-cultured for 16 h in liquid M9Glu+casAA media (*n*=4 biological replicates ±sem). Statistical significance was determined by two-tailed, unpaired *t*-test (*P<0.05, **P<0.005, ***P<0.0005.

**Fig. 7.**
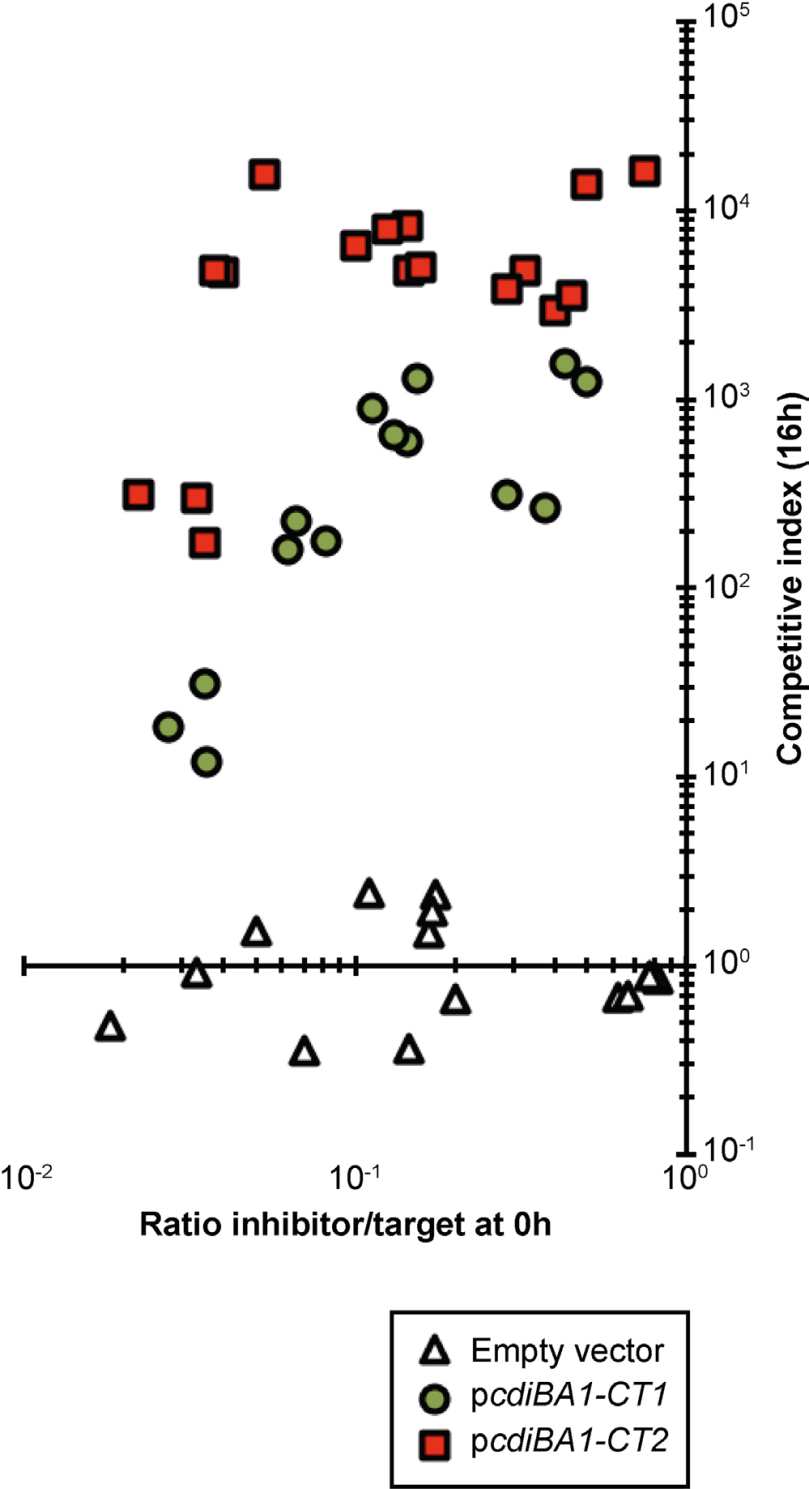
CdiA-CT2 is a more potent toxin than CdiA-CT1 at any given inhibitor-to-target cell ratio. (**a**) *E. coli* MG1655 inhibitor cells containing expression plasmids used to deliver either the CdiA-CT1 or CdiA-CT2 toxins, indicated in the lower box, were mixed with *E. coli* MG1655 target cells and co-cultured for 16 h in liquid M9Glu+casAA media. CIs (*y*-axis) were determined at different inhibitor to target ratios (*x*-axis). Each symbol represents one biological replicate.

In M9Glu+casAA medium, CdiA2 appeared to be as effective as CdiA1 in inhibiting the growth of target cells lacking the cognate CdiI immunity protein, while displaying an ≈4.5-fold lower expression compared to CdiA1 (Figs 3a, b and 5a–f). Thus, higher relative expression of CdiA1 versus CdiA2 did not result in a concomitant increase in growth inhibition. To explore the contribution of both CdiA expression levels and any differences in the toxic potency of CdiA-CT1 or CdiA-CT2, we used previously designed constructs in which *cdi-1* is expressed from a multicopy plasmid [4] or from the *E. coli* MG1655 chromosome [32]. To achieve a comparable level of CDI intoxication, mediated by either CdiA-CT1 or CdiA-CT2, and to account for any possible differences in CdiA1- and CdiA2-mediated toxin delivery (due to protein sequence differences, Fig. S2), we generated chimeric *cdiA1* constructs by replacing the native *cdiA*-CT1 and immunity region with the *cdiA-*CT2 and immunity region in either the chromosomally located *cdi-1* locus in MG1655 [32] or in the plasmid-borne *cdi-1* locus [4]. This resulted in both chromosomal- and plasmid-located *cdiA1*-CT/I2^EC93^ chimaeras (Fig. 6a). Chromosomal expression of *cdi-1* resulted in an approximately 14-fold lower CdiA expression level, compared to plasmid-borne *cdi-1* expression, and we could not identify any differential expression of CdiA1-CT1 compared to CdiA1-CT2 (Fig. 6b). *E. coli* MG1655 isolates expressing chromosomally encoded *cdi-1* outcompeted target cells by approximately 1.1-log of growth inhibition, whereas cells with a chromosomally encoded *cdiA1*-CT/I2^EC93^ chimeric construct outcompeted target cells by approximately 1.6-log of growth inhibition in liquid media (Fig. 6c). Inhibitor cells equipped with plasmid-borne *cdi-1* or *cdiA1*-CT/I2^EC93^ outcompeted the same targets by 2.4- and 3.6-log, respectively (Fig. 6c). Neither of the inhibitor strains outcompeted targets expressing the non-cognate BamA^Sty^ receptor (Fig. 6c). Together, these results indicate that higher levels of CdiA expression result in increased growth inhibition of target cells and that when delivered by the same CdiA protein, CdiA-CT2 is able to mediate a greater amount of growth inhibition compared to CdiA-CT1. Thus, CdiA-CT2 is a more potent toxin than CdiA-CT1. This discrepancy in toxic potency could account for the observation that both CdiA1 and CdiA2 in EC93 are equally as effective at inhibiting the growth of target cells, even though CdiA2 is expressed at about one-fifth the level of CdiA1 (Fig. 3a, b). This conclusion is further supported by our finding that inhibitor cells expressing the CdiA-CT2 toxin depolarized the membrane of a greater fraction of target cells during co-cultivation, compared to inhibitor cells expressing the CdiA-CT1 toxin (Fig. 4a–d). However, to further test this hypothesis we set up growth competitions in liquid M9Glu+casAA medium over a range of inhibitor:target cell ratios using plasmids expressing either the *cdi-1* locus or the *cdiA1*-CT/I2^EC93^ chimaera (Figs 6a and 7). Target cells co-cultured with inhibitors expressing the CdiA-CT2 toxin were always inhibited more, at any given inhibitor:target cell ratio, than target cells that were co-cultured with inhibitors expressing CdiA-CT1 (Fig. 7b). Together, these results support the conclusion that CdiA-CT2 is a more potent toxin than CdiA-CT1.

## Discussion

Here we show that the original isolate in which the phenomenon of CDI was discovered, *E. coli* EC93, employs two plasmid-borne *cdi* systems to inhibit the growth of susceptible bacteria *in vitro*. Both CdiA toxins deliver a C-terminal effector domain that dissipates the PMF, suggesting that they may form pores in the membrane [27]. However, although both toxins have similar activities, the potency of these toxins differs. This raises important questions about how the toxins mediate their growth inhibitory activities and what factors are important for CDI toxicity. Pore-forming colicins depolarize target cell membranes by spontaneously inserting into the lipid bilayer to form ion channels [33]. Although much is unclear about how pore-forming colicins form an open gate in the inner-membrane, all evidence suggests that colicin channels are monomeric (reviewed by Kienker and colleagues [34]), and yet they form pores that appear to be too large to model. A possible explanation for the difference in potency between the EC93 CdiA toxins could be that the minimal number of toxins required for toxicity is different for the two toxins. For example, multiple toxin molecules might be required to form a pore in the membrane and the number of toxin molecules required for this could differ for the two toxins. However, neither CdiA-CT1 nor CdiA-CT2 are active against target cells that lack the AcrB component of the AcrAB-TolC multidrug efflux pump [8], suggesting a mode of action that differs from that of pore-forming colicins. Thus, it is possible that the difference in toxicity is due to how the two toxins interact with AcrB. For example, the CDI effectors could mediate toxicity by opening up the AcrB proton channel, located in the inner membrane, rather than by forming pores by itself. Alternatively, the affinity by which the proteins interact with AcrB could be different for the two toxins. Further studies are required to find out if and how AcrB promotes membrane insertion of the CdiA toxins and the mechanism(s) by which these toxins collapse the PMF.

The *cdi-1* system in EC93 is expressed at a higher level than *cdi-2*. Our results indicate that, for each CDI system, higher levels of CdiA expression result in increased growth inhibition. Thus, the number of CdiA molecules (i.e. deliverable toxins) on an individual bacterium appears to modulate the ability of that cell to outcompete neighbouring non-immune cells. Another factor modulating growth competition is toxin potency. Although expression of *cdi-2* is significantly lower than expression of *cdi-1* in rich and in semi-defined media, the *cdi-2* locus effects a similar level of growth inhibition compared to the *cdi-*1 locus. Based on these results, expressing more toxin with a greater potency should give the bacterium an even higher competitive ability. Yet both CdiA-CT1 and CdiA-CT2 toxins provide a similar growth advantage, suggesting that there may be an optimal level of inhibition to be achieved for an individual *cdi* locus. It is possible that the fine-tuning of *cdi* expression in relation to CdiA-CT toxicity (i.e. that a more potent toxin is expressed at a lower level) could be important for self-delivery, rather than interbacterial competition. We have previously shown that delivery of CdiA-CT1 toxins in clonal bacterial populations functions as a bet-hedging strategy that increases stress tolerance in the bacterial population [32]. At high population densities some EC93 cells sense the CdiA1 toxin, resulting in growth arrest that allows these bacteria to survive antibiotic exposure, a phenomenon referred to as persistence. It is thus possible that if too much CdiA-CT2, which is a more potent toxin, is delivered between EC93 cells, the increased toxin exposure might cause permanent or transient damage to the cells. Alternatively, too many cells in the population might be affected, resulting in a suboptimal bet-hedging strategy.

Bacterial genomes are plastic in terms of gene content. Horizontal gene transfer allows influx of genes, which remain in the genome if (i) they provide the bacterium with a fitness benefit or (ii) their loss is not associated with a fitness cost. Plasmid-encoded genes are readily transferred between bacteria. Here we find two *cdi* loci on a low-copy plasmid, both expressed and providing EC93 with a competitive advantage against other *E. coli* strains under laboratory conditions. A rough estimate of the location of *E. coli *-specific *cdi* systems in fully sequenced genomes indicates that while there are *cdi* systems located on plasmids (such as in EC93), most are chromosomally located. Many of these chromosomally located systems seem to be highly regulated, and not expressed under most laboratory conditions [18, 35, 36]. Thus, the high-level expression of the EC93 *cdi-1* system in laboratory media seems to be rather unusual. Furthermore, the presence of more than one *cdi* system on an *E. coli* plasmid seems to be exceedingly rare, with the pCP127 plasmid appearing to be the only known example of such a plasmid.

A large portion of pCP127 (~47 kb), including the *oriV* and parts of *cdiBA2* (but not the CdiA-CT2 toxin and immunity region found in EC93), are identical to a previously identified plasmid (Plasmid A) found in an *E. coli* strain isolated from pigs (GenBank accession no.: CP010207.1). This strain also contained a second, mobilizable plasmid (Plasmid B) and 49 kb of this plasmid is homologous to the pCP127 plasmid, while the remaining 31 kb of pCP127 contains the *cdi-1* locus (Fig. S10). The pCP127 plasmid lacks genes mediating horizontal gene transfer and an *oriT* and seems to be the result of a recombination event between these two plasmids (A and B) in the pig isolate and presumably a third recombination, and/or duplication event, adding the *cdi-1* region. Previous results showed that insertion of *cdi-1* on a mini-F plasmid provided strong selection for maintenance of the plasmid via a surveillance mechanism [37]. It is therefore likely that the two *cdi* systems stabilize the pCP127 plasmid in *E. coli* strains, but not in other species because both *cdi* systems are *E. coli *-specific, targeting *E. coli *-specific residues in the extracellular loops of BamA [23, 37]. Moreover, the pCP127 plasmid contains 13 kb homology to the EC93 chromosome, suggesting that it may integrate into the genome of EC93, which would further increase the stability of the plasmid in EC93.

EC93 was originally isolated from a rat where it dominated the *Enterobacteriaceae* flora [4]. Although the phenomenon of CDI was discovered using EC93, little was known about this *E. coli* isolate. Based on the whole-genome sequencing, bioinformatic and functional analyses described here, we identify a number of fitness and competition factors that might explain its ability to dominate in the normal flora. It will be important to determine how CDI toxins and the T6SS contribute to the ability of EC93 to compete with other bacteria *in vivo*, and under what conditions these systems are active. The genome of EC93 contains an *iss* gene, a virulence factor often found in pathogenic Shiga toxin-producing *E. coli* (STEC), while the pCP127 plasmid contains virulence factors (e.g. K88 fimbrial genes) normally found in enterotoxigenic *E. coli* (ETEC). This might indicate that the genomic ancestor of EC93 was a STEC and that the pCP127 plasmid originated from an ETEC, but was transferred to EC93, possibly through a distant horizontal gene transfer event. The impact of these virulence factors on the *in vivo* lifestyle and/or growth behaviour of EC93 is still unknown. However, this melting pot of potential virulence factors and other important fitness factors makes EC93 an attractive organism to study and a good potential model organism for both CDI and T6SS research.

## Supplementary Data

Supplementary material 1

Supplementary material 2
