# Supplementary material for: Escherichia coli EC93 deploys two plasmid-encoded class I contact-dependent growth inhibition systems for antagonistic bacterial interactions
Source: Microb Genom. 2021 Mar 1;7(3):mgen000534. doi: 10.1099/mgen.0.000534 (PMC8190604; doi:10.1099/mgen.0.000534)
Supplement: Supplementary material 1 [file mgen-7-0534-s001.pdf]

Supplementary material for:

E. coli EC93 deploys two plasmid-encoded class I CDI systems for antagonistic bacterial interactions

Marcus Wäneskog[1], Tiffany Halvorsen[2], Klara Filek[1,3], Feifei Xu[1], Disa Hammarlöf[1,4], Christopher S. Hayes[2], Bruce A. Braaten[2], David A. Low[2], Stephen J. Poole[2], and Sanna Koskiniemi[1*]

Affiliations

[1] Department of Cell and Molecular Biology, Uppsala University, Sweden.

[2] Department of Molecular, Cellular and Developmental Biology, University of California Santa Barbara, USA

[3] Current address: Department of Biology, University of Zagreb, Croatia.

[4] Current address: Science for Life Laboratory, KTH, Sweden.

* Correspondence to: sanna.koskiniemi@icm.uu.se

This supplementary file includes:

Supplementary methods

Chromosomal and plasmid constructs

Supplementary tables S1-S6

Supplementary figures S1-S10

Supplementary methods

Minimal inhibitory concentration (MIC) measurements

MIC assays were carried out using E-test according to the instructions of the manufacturer (AB bioMerieux, Solna, Sweden). E-tests were performed on LB agar plates that were incubated for 16–18h at 37°C.

Prophage identification

The EC93 genome was analyzed for the presence of intact prophages and partial prophage sequences using PHASTER (PHAge Search Tool Enhanced Release) [1, 2].

Phage plaque assay

Cultures of EC93 were grown overnight in LB liquid broth supplemented with 10mM $MgSO_4$ and 5mM $CaCl_2$. Cultures were then diluted 1:10 in fresh LB supplemented with 10mM $MgSO_4$ and 5mM $CaCl_2$ and 5mL were aliquoted in an empty petri dish and subjected to varying degree of UV light (254 nm). After UV exposure cells were left to recover for 2 h at 37°C to allow for prophage induction. Cultures were then pelleted at 3000xg for 5min and the supernatant was filtered through a 0.2µm filter before being spotted on a soft-agar plate (top-agar 0.5%, bottom agar 1.5%) made with a lawn of *E. coli* MG1655.

Transposon mutagenesis to identify EC93 CdiA-CT2 resistant mutants

To generate CDI-resistant mutants, target cells were mutagenized, mixed with CDI^+ inhibitor cells, and CDI-resistant mutants were selected as described previously [3]. The mariner transposon [4] was introduced into *E. coli* DL8705 by conjugation with MFD*pir* cells carrying plasmid pSC189. Donors were supplemented with 30µM diaminopimelic acid in shaking liquid LB and grown to mid-log at 37°C. Donors and recipients were mixed and plated on LB agar at 37°C for 5 h. Cell mixtures were harvested with a sterile swab, collected in 1×M9 medium, and plated on kanamycin-supplemented LB agar for selection of transposon integrants. Each mutant pool was harvested into 0.5 mL 1×M9 media for selection. Each mutant pool was subjected to selection by co-culture with EPI100 *Δwzb* carrying plasmid pDAL879-*cdiA1-CT2/cdiI2*. The surviving colonies were collected again into 1 mL 1×M9 media and subjected to two more rounds of co-culture selection. Transposon insertion sites were determined by rescue cloning. Chromosomal DNA from each resistant mutant was

digested overnight with NspI at 37°C followed by a 20 min inactivation step at 65°C. Each reaction was supplemented with ATP and T4 Ligase and incubated overnight at 16°C. The reactions were electroporated into *E. coli* DH5α *pir*$^+$ cells. Plasmids from the resulting kanamycin-resistant colonies were isolated and transposon insertion junctions identified by sequencing using primer CH2260.

CdiA SDS-PAGE assay

MG1655 cells expressing CdiA from the chromosome or from a medium-copy plasmid (table S4) were diluted 1/1000 from an overnight culture and grown to logarithmic phase (OD_{600} = 0.4). Bacterial cultures (1 mL) were pelleted at 21,000 x g for 10 min and re-suspended in 100 µl of a 1x membrane-protein sample buffer [50mM Tris pH 6.8, 1% SDS (w/v)], 1% Triton X-100, 10% Glycerol, 0.2% bromophenol blue) and boiled for 5 min at 95°C. DTT (150 mM) was added to each sample before pelleting at 21,000 x g for 5 min, then supernatant were loaded and analyzed by SDS-PAGE (NuPAGE Tris-Acetate 3-8% gel, Thermo Scientific, USA). Total protein was detected *in situ* using SYPRO Ruby Protein Gel Stain (Thermo Scientific, USA) and visualized by UV light (254 nm). Relative protein abundance was calculated by densitometry using ImageJ.

Chromosomal and plasmid constructs

Construction of EC93 cdi mutants

The *cdi-1* and *cdi-2* loci were deleted by amplifying a *catR* selection cassette [5] using primers 1185/1273, for *cdi-1*, and primers 767/1186 for *cdi-2*, and selecting for recombinants using the phage λ Red system [6]. To prevent auto-inhibition of growth during the KO of *cdi-1*, prior to recombineering, EC93 was first transformed with the temperature-sensitive plasmid pSK2757 expressing cognate CdiI1 immunity, which was subsequently cured from EC93 by growing cells at 42°C. Plasmid pSK2757 was constructed by amplifying the *cdiA1* toxin-immunity region with primers 543/544, then blunt-end ligating this fragment to a pSC101 vector backbone generated by amplification using primers 477 and 921. To prevent auto-inhibition during the KO of *cdi-2*, prior to recombineering, EC93 was transformed with plasmid pSK1244 expressing cognate CdiI2 immunity. Plasmid pSK1244 and pSIM27 [7] both share a

pSC101 origin of replication, but have distinct antibiotic selection markers (kanR and tetR respectively). Plasmid pSK1244 was segregated out of the population by only selection for pSIM27 after lambda red recombineering. Plasmid pSK1244 was constructed by amplifying the *cdiA2* toxin-immunity region with primers 547/548, then blunt-end ligating this fragment to a pSC101 vector backbone generated by amplification using primers 477 and 921. The constructs were verified by PCR using primers 1489 with 1502, 1491 with 630 and 564 with 1491.

Construction of EC93 catR mutant

An chloramphenicol resistant EC93 strain was created by inserting a catR selection cassette [5] downstream of the *cdi-1* locus in EC93 by amplifying the catR cassette using primers 1500/1501 and selecting for recombinants using the phage lambda Red system [6]. The construct was verified by PCR using primers 1053 with 1502.

Construction of EC93 cdiB-sYFP2 reporter constructs

The *sYFP2* reporter cassette was amplified from plasmid pSK2991 with primers 876 and 877. The resulting PCR product was integrated into the *cdi-1 and cdi-2* loci in either a EC93 Δ*cdi-1 or* Δ*cdi-2* strain by lambda red recombination [6]. The constructs were verified by PCR using primers 1489 and 1490, as well as primers 1491 and 1492.

Construction of acrB mutant

An *acrB* mutant strain (CH14008) was generated by transducing the *acrB::kanR* cassette from the Keio deletion strain into CH7175.

Construction of acrB expression plasmid

The *acrB* gene was amplified from *E. coli* MG1655 genomic DNA with primers CH4498 and CH4355, digested with BamHI and XbaI and ligated into plasmid pZS21.

Construction of pCloDF1(PJ23109-cdiI2) expression plasmid

The pCDF::*pJ23101-cdiI1*^{EC93} expression plasmid [8] was modified to replace the strong pJ23101 promoter with the weaker pJ23109 promoter as follows. The *cdiI2*

gene was amplified from EC93 genomic DNA using primers 1117 and 1118, then ligated to the DNA fragment resulting from PCR-amplification of plasmid SK1757 with primers 1198 and 1200. This resulted in placement of the *cdiI2* gene under control of the PJ23109 promoter. The resulting plasmid was verified by sequencing using oligonucleotides 986 and 987.

Construction of chimeric cdiA1-CT2 chromosomal insertion.

The EC93 *cdiA*-CT/I1 region of the *cdiBAI1* locus of EC93, previously inserted down-stream of *lacA* in the chromosome of MG1655 [11], was replaced with the EC93 *cdiA*-CT/I2 region of locus 2, at the conserved VENN motif. This was done by PCR amplifying the EC93 *cdiA*-CT/I2 with primers 1862 and 1863 and a kanamycin resistance cassette [5], using primers 1864 and 1865. Simultaneously inserting 40bp homology to the *cdi-1* locus and to each PCR fragment. The PCR products were fused by overlap-extension PCR (OE-PCR) using primers 1862 and 1865. The resulting OE-PCR product was transformed into SK4790 by lambda red recombination [6]. The construct was verified by PCR and sequenced using primers 1057 with 564.

Construction of chimeric cdiA1-CT2 expression plasmid

The EC93 *cdiA*-CT/I1 region from *cdi-1* was replaced with the EC93 *cdiA*-CT/I2 region of locus 2, at the conserved VENN motif. This was done using cosmid recombineering with PheS* counter-selection using plasmid pCH10163, as previously described [9]. EC93 *cdiA*-CT/I2 was amplified by PCR with primers CH3822 and CH3823 and fused to upstream and downstream homology regions amplified from EC93 *cdiA* locus 1 using primer pairs DL1527 and DL2470 or DL1663 and DL2368, respectively. The PCR products were fused by overlap-extension PCR (OE-PCR) using primers DL1527 and DL2368. The resulting OE-PCR product was transformed along with pCH10163 into *E. coli* DY378 cells, as previously described [9]. The resulting recombinants were selected on yeast extract glucose agar containing 33 µg/mL chloramphenicol and 10 mM D/L-p-chlorophenylalanine.

Table S1. Bacterial strains used in this study

Strain number	Genotype	Origin
CH7175	EPI100 Δ*wzb*	This study
CH7176	EPI100 Δ*wzb*::*kanR*	This study
CH14008	EPI100 Δ*wzb*, Δ*acrB*	This study
DL3717	*E. coli* from Charles River K93 Rat colony (EC93 WT)	[10]
DL8705	*E. coli* MG1655 *Δwzb, araBAD::spmR*	This study
SK431	*E. coli* K12 MG1655 /pDAL660Δ1-39	[11]
SK1455	*E. coli* MG1655 *Δwzb, ara::spec, galK::cat-RFP*	This study
SK2540	*E. coli* MG1655 *bamA*^{LT2}	[11]
SK2661	*E. coli* MG1655 *lacA-cdiBAI1-kanR*	[11]
SK2725	EC93 /pSC101(ts)::*cdiA*-CT/I1^{EC93} (RepA A56V mutation)	This study
SK2773	EC93 /pSC101(ts)::*cdiA*-CT/I1^{EC93} (RepA A56V mutation) /pSIM27	This study
SK2837	EC93 *cdiAI1::catR*	This study
SK2968	*E. coli* EPI100 *Δwzb* /pDAL660Δ1-39 (*cdiBAI1*^{EC93})	This study
SK2970	*E. coli* EPI100 *Δwzb* /pDAL660Δ2-63::*cat*	This study
SK2971	*E. coli* EPI100 *Δwzb* /pSK2971 (*cdiBA1*-CT/I^{NC101})	This study
SK2974	*E. coli* EPI100 *Δwzb* /pSK2974 (*cdiBA1*-CT/I2^{EC93})	This study
SK3022	*E. coli* MG1655 *lacA-cdiBA1(*ΔCT+I)	[11]
SK3099	EC93 DEL*cdiAI1*	This study
SK3290	*E. coli* MG1655 *Δwzb, ara::specR, galK::cat-RFP* /pTrc99 (Empty)	This study
SK3291	*E. coli* MG1655 *Δwzb, ara::specR, galK::cat-RFP* /pTrc99::*cdiI1*^{EC93}	This study
SK3292	*E. coli* MG1655 *Δwzb, ara::specR, galK::cat-RFP* /pTrc99::*cdiI2*^{EC93}	This study
SK3295	*E. coli* MG1655 *Δwzb, ara::specR, galK::cat-RFP* /pTrc99::*cdiI*$^{E.\ coli\ NC101}$	This study
SK3303	*E. coli* MG1655 *Δwzb ara::spe,c galK::cat-RFP, ΔacrB::kan*	This study
SK3935	*E. coli* MG1655 *rpsL* (K42R)	This study
SK3936	*E. coli* MG1655 *bamA*^{LT2}, *rpsL* (K42R)	This study
SK3954	*E. coli* MG1655 *rpsL* (K42R) /pCDF1::p*J23101*	This study
SK3955	*E. coli* MG1655 *rpsL* (K42R) /pCDF1::*pJ23101-cdiI1*	This study
SK3956	*E. coli* MG1655 *rpsL* (K42R) /pCDF1::*pJ23109-cdiI2*	This study
SK4062	EC93 *cdiAI1::catR, cdiB2-sYFP2-kanR*	This study
SK4096	*E. coli* MG1655 /pDAL660Δ2-63::*cat*	This study
SK4097	*E. coli* MG1655 /pDAL660Δ1-39 (*cdiBAI1*^{EC93})	This study
SK4098	*E. coli* MG1655 /pSK2974 (*cdiBA1*-CT/I2^{EC93})	This study

SK4464	EC93 /pSIM27	This study
SK4465	EC93 *DELcdiAI1* /pSIM27	This study
SK4466	EC93 /pSIM27 /pSC101::*cdiA*-CT/I2^{EC93}	This study
SK4467	EC93 *DELcdiAI1* /pSIM27 /pSC101::*cdiA*-CT/I2^{EC93}	This study
SK4485	EC93 *cdiBAI1-orphan* CT/I-*catR*	This study
SK4487	EC93 *DELcdiAI1, cdiBAI2::catR*	This study
SK4492	EC93 *cdiBAI2::catR*	This study
SK4493	EC93 *cdiBAI2::catR, cdiB1-sYFP2-kanR*	This study
SK4545	EC93 *cdiBAI1-orphan* CT/I-*catR* /pCDF1::p*J23101*	This study
SK4546	EC93 *cdiBAI1-orphan* CT/I-*catR* /pCDF1::*pJ23101-cdiI1*^{EC93}	This study
SK4547	EC93 *cdiBAI1-orphan* CT/I-*catR* /pCDF::(*pJ23109-cdiI2*^{EC93}	This study
SK4548	EC93 *cdiAI1::catR* /pCDF1::p*J23101*	This study
SK4549	EC93 *cdiAI1::catR* /pCDF1::*pJ23101-cdiI1*^{EC93}	This study
SK4550	EC93 *cdiAI1::catR* /pCDF1::*pJ23109-cdiI2*^{EC93}	This study
SK4551	EC93 *cdiBAI2::catR* /pCDF1::p*J2310*	This study
SK4552	EC93 *cdiBAI2:: catR* /pCDF1::*pJ23101-cdiI1*^{EC93}	This study
SK4553	EC93 *cdiBAI2:: catR* /pCDF1::*pJ23109-cdiI2*^{EC93}	This study
SK4554	EC93 *DELcdiAI1, cdiBAI2::catR* /pCDF1::p*J2310*	This study
SK4555	EC93 *DELcdiAI1*, *cdiBAI2::catR* /pCloDF1::*pJ23101-cdiI1*^{EC93}	This study
SK4556	EC93 *DELcdiAI1, cdiBAI2::catR* /pCloDF1::*pJ23109-cdiI2*^{EC93}	This study
SK4790	*E. coli* MG1655 *lacA-cdiBA1(*ΔCT+I) /pSIM27	This study
SK4791	*E. coli* MG1655 *lacA-cdiBA1*-CT/I2^{EC93}*-kanR*	This study

Table S2. Plasmids used in this study

Plasmid	Genotype	Origin
pSK917	pSC101 Empty	This study
pSK1244	pSC101::*cdiA*-CT/I2^{EC93}	This study
pSK1751	pCDF::pJ23101	[8]
pSK1752	pCDF1::pJ23101-*cdiI1*^{EC93}	[8]
pSK2350	pSC101(ts) Empty (RepA A56V mutation)	This study
pSK2351	pSC101(ts)::*cdiA*-CT/I1^{EC93} (RepA A56V mutation)	This study
pSK2902	pCDF::*pJ23109-cdiI2*^{EC93}	This study
pCH10163 (*cdiBA::kan-pheS*)	pDAL660Δ2-63::cat that carries a *kan-pheS** cassette in place of the *E. coli* EC93 *cdiA-CT/cdiI* coding sequence.	[9]
pDAL660Δ1-39 (*cdiBAI1*^{EC93})	pWEB containing a 16,734bp region from EC93 including the *cdiBAI1* locus	[10]
pDAL660Δ2-63::*cat*	pDAL660Δ1-39 with a *cdiBAI1* deletion	[10]
pSK2974 (*cdiBA1*-CT/I 2^{EC93})	pDAL660Δ1-39 with *cdiA1-CT1* and *cdiI1* replaced by *cdiA-CT2* and *cdiI2*	This study
pSK2971 (*cdiBA1*-CT/I^{NC101})	pDAL660Δ1-39 with *cdiA1-CT1* and *cdiI1* replaced by *cdiA-CT* and *cdiI* from *E. coli* NC101	[3]
pSIM27	Lambda red genes under thermal induction	[7]
pZS21-*acrB*	Derivative of pZS21 with constitutive expression of AcrB from *E. coli* MG1655	This study
pSC189	Mobilizable plasmid with R6Kγ replication origin; carries the mariner transposon containing kanamycin resistance cassette	[4]

Table S3. Oligos used in this study

**Oligo**	**Sequence (5' to 3')**
274	ATCGATAAACCTCACTTGTTGAAGTGCCATTAACGTCAACCCATATGAA<br>TATCCTCCTTAGTTCC
275	CATATTCACTACCGCCAAGTAAATTATTCTCAACAACAAACCATATGAA<br>TATCCTCCTTAGTTCC
477	GCTCGAGGTGAAGACG
543	TAGTTAGTTAGATTAGTTGAGAATAATGCAATGTCTGG
544	CATAGTTTTCATCATTCGCGGCTTTTCTGTCTAAGATACTAAGGCCC
547	TAGTTAGTTAGATTAGTTGAGAATAACTCACTCAGTGATG
548	CATAGTTTTCATCATTCGCGGCACGATAAAAACGATTTAATATCAATAT<br>G
564	GTGTTCCGCTTCCTTTAGCAG
630	ACCATACTATATCGACCAAG
767	GGCGCGGTTCTCCGTGGATATGCTATCTCTCCCTCTCCGTTGTAGGCTGG<br>AGCTGCTTC
876	ATCTCACGGTTTACTGGCGCGTTGCCGTCGCGTTTTAAAGAGAAAGAGG<br>AGAAATACTAG
877	GAAGCGAACGGGAGGCTGATGCATGGTAATAATCCCCATATGAATATC<br>CTCCTTAGTTCC
921	TTCAGAGAAAGAGGAGAAATACTAG
986	ATCACCACCACCATCACGTGG
987	GTGACCGTGTGCTTCTCAAATGC
1053	GCAAGATGTGGCGTGTTACG
1057	CCATTGCTTCAGGTATTGCAGG
1117	ATAAGGATCCCAACAAGTGAGGTTTATCGATATG
1118	TACAGTCGACAATTATTCTCAACAACAAATCAACG
1185	TTACTGGCGCGTTGCCGTCGCGTTTTAAGGGATTATTACCTGTAGGCTG<br>GAGCTGCTTC
1186	CCATGCTGGCGTATCCACGCAGCATGAGCATATTCACTACCTTAGTTCC<br>TATTCCGAAGTTCC
1198	TTTACAGCTAGCTCAGTCCTAGGGACTGTGCTAGCCTCGAGACTTGATA<br>TCGCGTGG
1200	TGAGCGCAACGCAATTAATGTAAG
1273	GAGCAGCAACCGCACAGCCTCTTGCTATCAAGCTCAGCGACTTAGTTCC<br>TATTCCGAAGTTCC
1489	GATAATCGCACTGACCAGGTAAC
1490	AGTGACTGGAATCATCGTTATTGC
1491	GGCAGAACAACATTCTGACG
1492	GGTCATCTCTGTCAGAGTGC

1500	TATGGATATAGCAAAAATAGATAAGCTATCTAATGCCTAATGTAGGCTG GAGCTGCTTC
1501	GCGAAGCAAAAAATCCCGACTTTGAGACCAGAATATCTATCCATATGA ATATCCTCCTTAGTTCC
1502	GGTGAGAAGCAGGCTATC
1862	AAGTGCTGCCGGTACGGGGGCACAGGCAGGTAGGAACTCGGTTGAGAA TAACTCACTCAGTG
1863	TTGCGCCCTGAGTGCTTGCGGCAGCCAACAAATCAACGATAAAAACG
1864	AATCGTTTTTATCGTTGATTTGTTGGCTGCCGCAAGCACTCAGGG
1865	GTCGTTAATCCATGCCCGATTATTCAATGTTCTGTGTCCAGAAGAACTC CAGCATGAGATCC
CH2260	CAAGCTTGTCATCGTCATCC
CH2131	TGCAGATCTTGTGGCAACAATTTCTAC
CH2132	GCGACCCGGGTGTATCAAACAAGACG
CH3822	CAGGTAGGAACTCGGTTGAGAATAATTCACTCAGTGATGGCTGGAAC
CH3823	GGTCTGGTGTCTAACCTTTGGGTTAACGATAAAAACGATTTAATATCAA TATGATGA
DL1527	GAACATCCTGGCATGAGCG
DL1663	CCCAAAGGTTAGACACCAGACC
DL2368	GTTGGTAGTGGTGGTGCTG
DL2470	ATTATTCTCAACCGAGTTCCTACCTG
CH4355	TTTGAGCTCTAGAATCAATGATGATCGACAGTATGGC
CH4498	TTCGGATCCGTTAAGACATGCC

Table S4. Predicted virulence factors and competition systems in the genome of EC93

Predicted fitness factors on the chromosome	Description
gad	Glutamate decarboxylase
iss	Increased serum survival
ipfA	Long polar fimbriae
fimH38	Type 1 fimbriae
T6SS locus	Type VI secretion system
3 *vgrG* genes	T6SS component
8 *rhs* genes	T6SS toxic effector protein
mdfA	Multidrug efflux pump

Predicted fitness factors on the pCP127 plasmid	Description
pic	Serine protease auto-transporter
K88 fimbriae locus	Aggregation protein
RTX family exoprotein A	Putative virulence protein
2 *cdiBAI* loci	T5SS, subclass b

Table S5. MIC (μg/mL) of EC93 and MG1655 towards antibiotics used in this study

	**EC93**	***E. coli* MG1655**
Streptomycin	8	6
Chloramphenicol	8	3
Kanamycin	8	8

Table S6. EC93 phage release after exposure to UV light (254 nm). Plague assay was performed on *E. coli* MG1655 (n=3).

UV light (mJ/cm2)	Plagues
0	0
25	0
50	0
100	0
200	0
400	0
600	0
800	0
1000	0

Table S7. Chromosomally located intact *E. coli*-like *cdi* systems.

List of all 177 *E. coli cdi* systems identified to be present in 133 unique bacterial genomes by blastn search, using the *cdiA1* gene from EC93 as query.

(See supplementary excel file, available with the online version of this paper.)

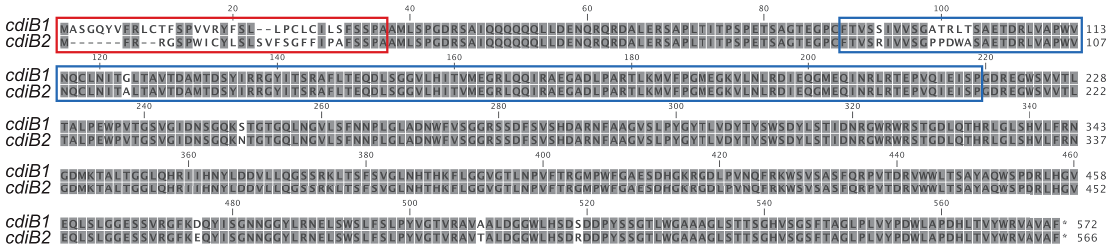

Figure S1. Alignment of CdiB1 and CdiB2 proteins from *E. coli* EC93.

Identical residues are shown in grey and non-identical residues are white. Sequences boxed in red encompass the putative SEC-signal peptide and sequences boxed in blue encompass the putative POTRA domains.

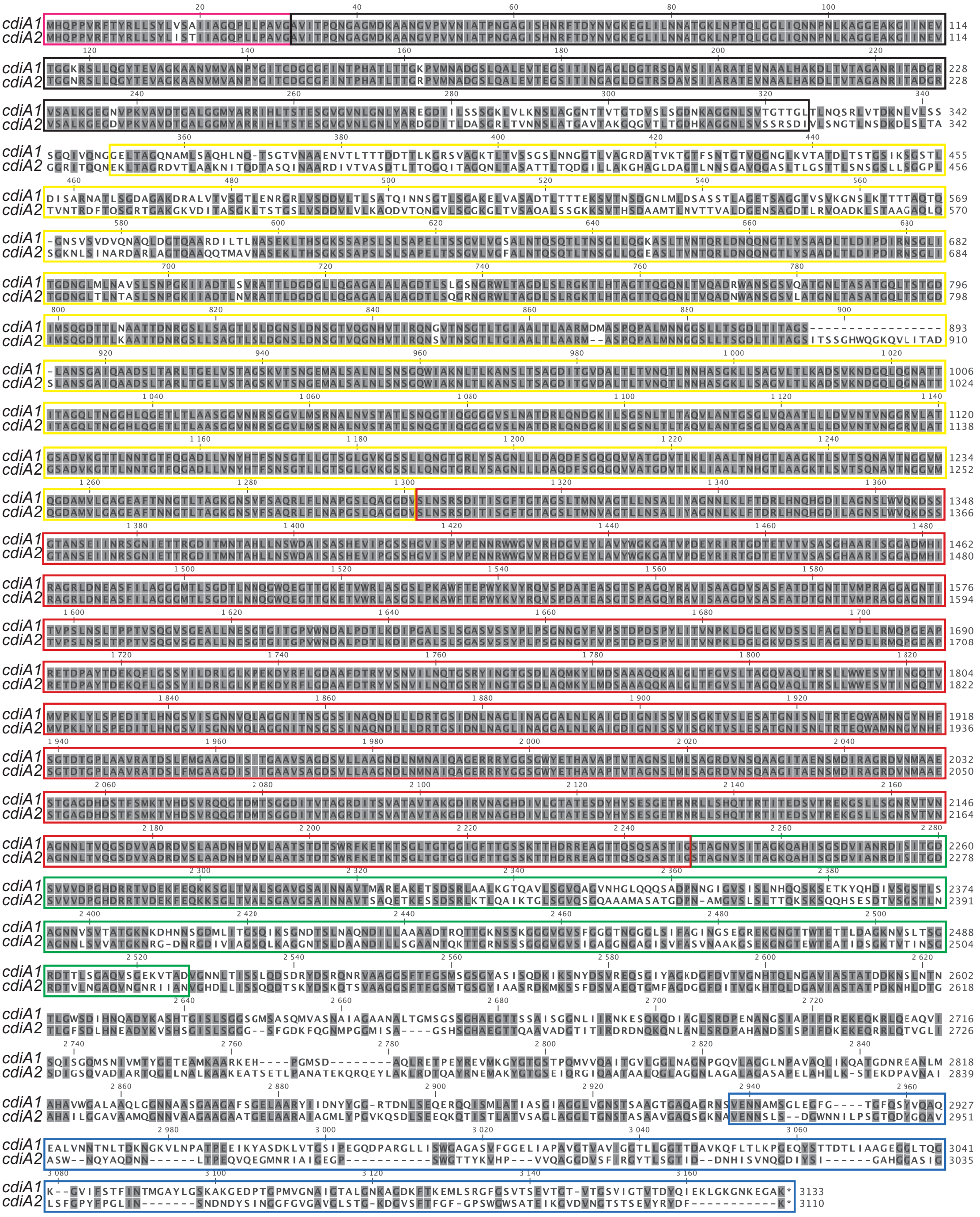

Figure S2. Alignment of CdiA1 and CdiA2 proteins from EC93.

Identical residues are shown in grey and non-identical residues are white. Sequences boxed in pink encompass the putative SEC-signal peptide, sequences boxed in black encompass the Two-Partner Secretion domain (TPS), sequences boxed in yellow encompass the FHA-1 domain, sequences boxed in red encompass the extended Receptor-Binding Domain (RBD) region (class I), sequences boxed in green encompass the FHA-2 domain and sequences boxed in blue encompass the C-terminal domain containing toxin.

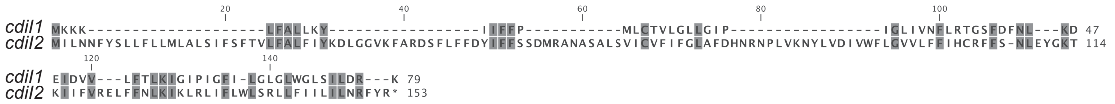

Figure S3. Alignment of CdiI1 and CdiI2 proteins from EC93.

Identical residues are shown in grey and non-identical residues are white.

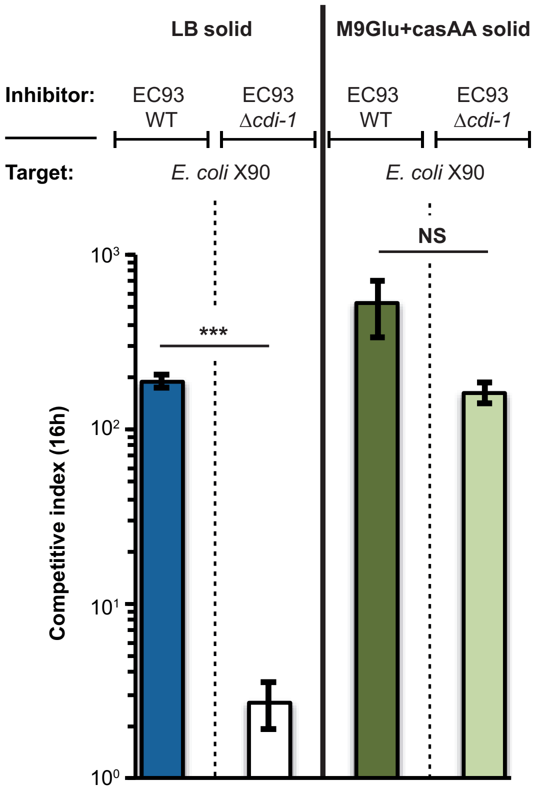

Figure S4. The growth inhibitory activity of the *cdiBAI2* locus of EC93 was not identified because the system is not active on LB media.

Average competitive index (CI) of wild-type EC93 and EC93 *Δcdi-1* cells co-cultured with *E. coli* X90 for 16h on LB solid medium or M9Glu+casAA solid medium (n=3 biological replicates ±SEM). Competitions were performed as previously described [12]. Statistical significance was determined by two-tailed, un-paired t-test (p-value: ***=<0.0005).

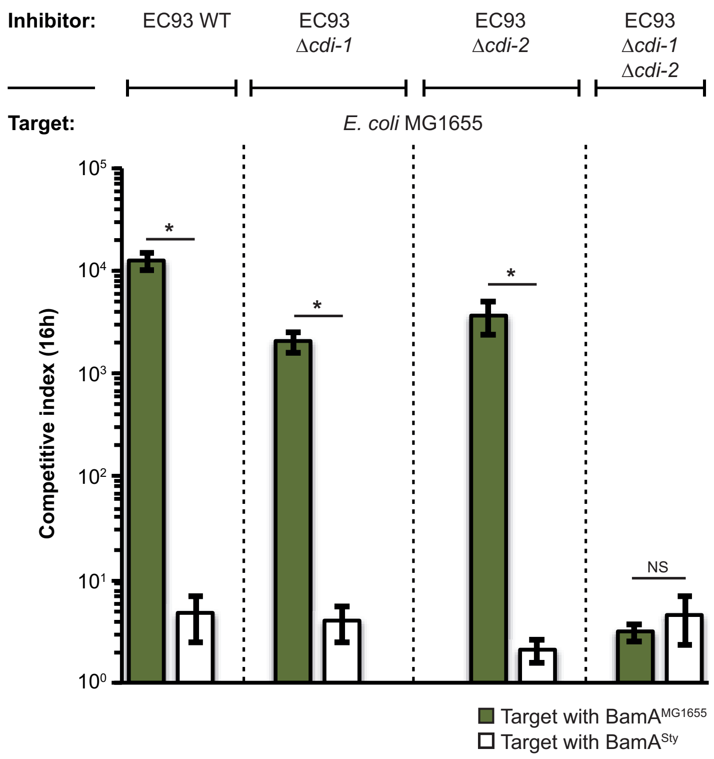

Figure S5. EC93 CdiA1 and CdiA2 use BamA as a receptor.

EC93, EC93 *Δcdi-1,* EC93 *Δcdi-2* or EC93 *Δcdi-1 Δcdi-2* cells were co-cultured with MG1655 cells expressing *E. coli* BamA (BamA^{MG1655}) or MG1655 expressing *Salmonella* BamA (BamA^{Sty}). Growth competitions were carried out for 16 h on M9Glu+casAA solid media (n=3 biological replicates ±SEM). Competitive indices are shown on the y-axis. Statistical significance was determined by two-tailed, unpaired t-test (p-value: *<0.05).

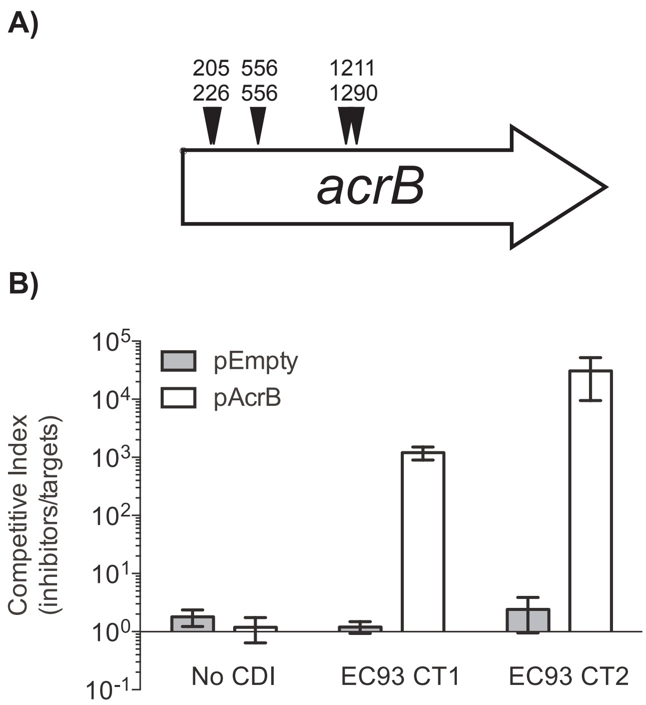

Figure S6. EC93 CdiA1 and CdiA2 both require AcrB for inhibition of target cells.

A) Schematic representation of mariner transposon insertions in *acrB* that conferred resistance to the Cdi-2 system (see Materials and Methods). B) Dependence of the *cdi-2* system on AcrB for growth inhibitory activity. EPI100 inhibitor cells expressing plasmid-encoded Cdi-1 delivering either the CdiA-CT1 toxin or the CdiA-CT2 toxin were co-cultured with EPI100 *ΔacrB* target cells transformed with an empty pZS21 vector control or a pZS21-AcrB expression vector. Competitions were carried out for 24h on LB solid media (n=3 biological replicates ±SEM). Competitive indices are shown on the y-axis.

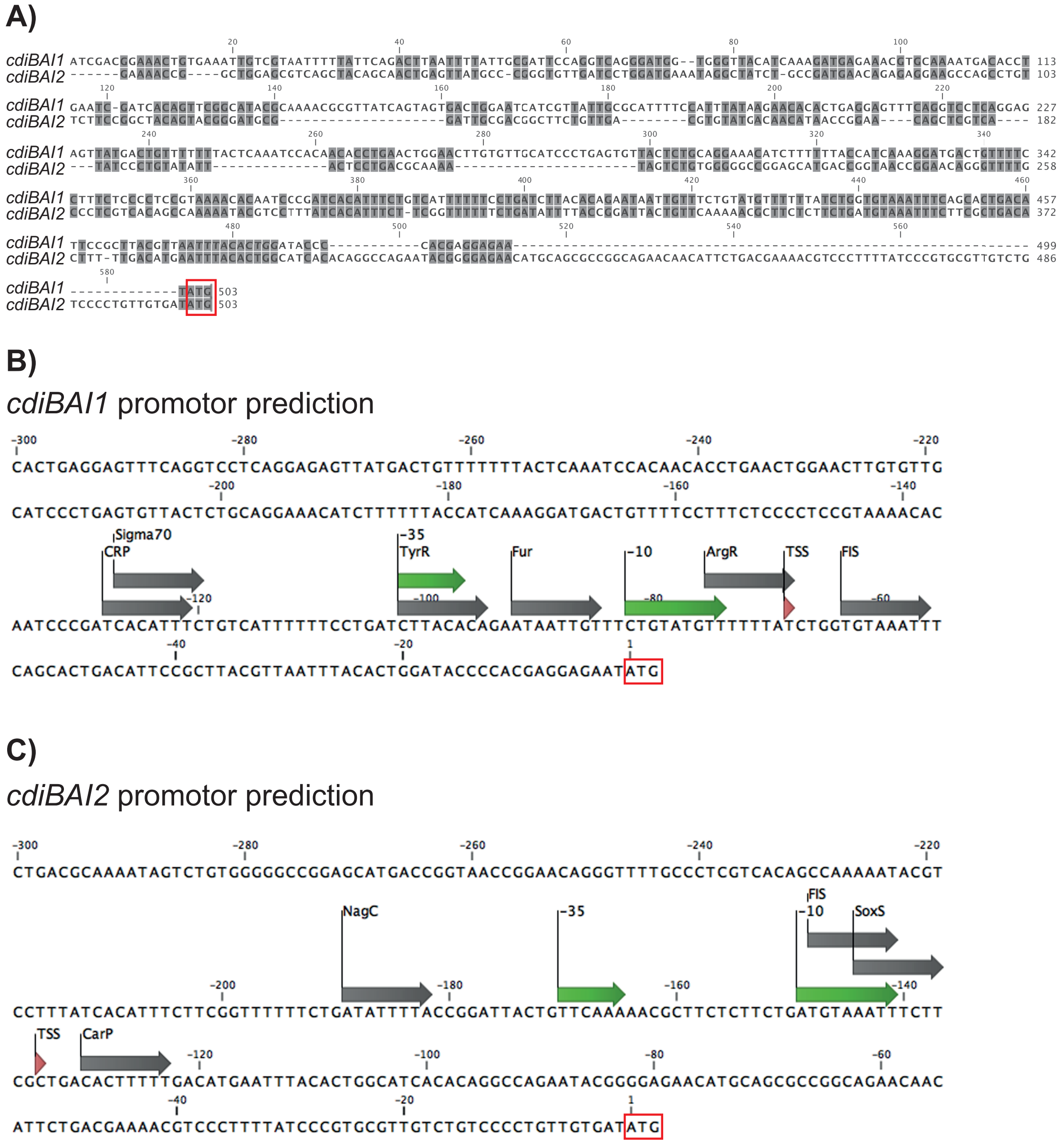

Figure S7. The *cdiBAI1* and *cdiBAI2* promoter regions.

A) Alignment of the *cdiB1* and *cdiB2* upstream regions (500bp). Identical residues are shown in grey and non-identical residues are white. The DNA sequences encoding *cdiB* AUG start codons are indicated by a red-box. B, C) Putative promoter predications of the two *cdiBAI* loci promoters of EC93. Red-arrows indicate the transcriptional start-site (TSS). Grey-arrows indicate putative binding-sites for transcriptional regulators. Green-arrows indicate the location of the putative -10 and -35 motifs and the AUG start codons of *cdiB1* and *cdiB2* are indicated by a red-box.

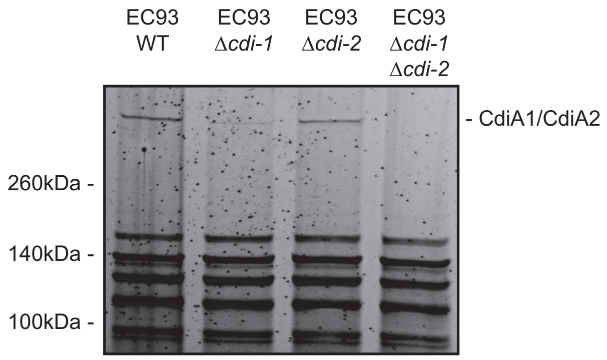

**Ratio:**	≈1	≈0.20	≈0.71	ND
**Ratio (WT vs. A1+A2):**	≈1	≈0.91		
**Ratio (A1 vs. A2):**		≈1	≈3.53	

CdiA1 ≈319kDa
CdiA2 ≈316kDa

Figure S8. EC93 CdiA1 and CdiA2 are differentially expressed.

The relative expression of the *cdi-1* or *cdi-2* loci in EC93 was analyzed by SDS-PAGE after cells were grown in M9-Glu+casAA media. Total protein was visualized by SYPRO Ruby staining. Relative protein abundance was calculated by densitometry using ImageJ.

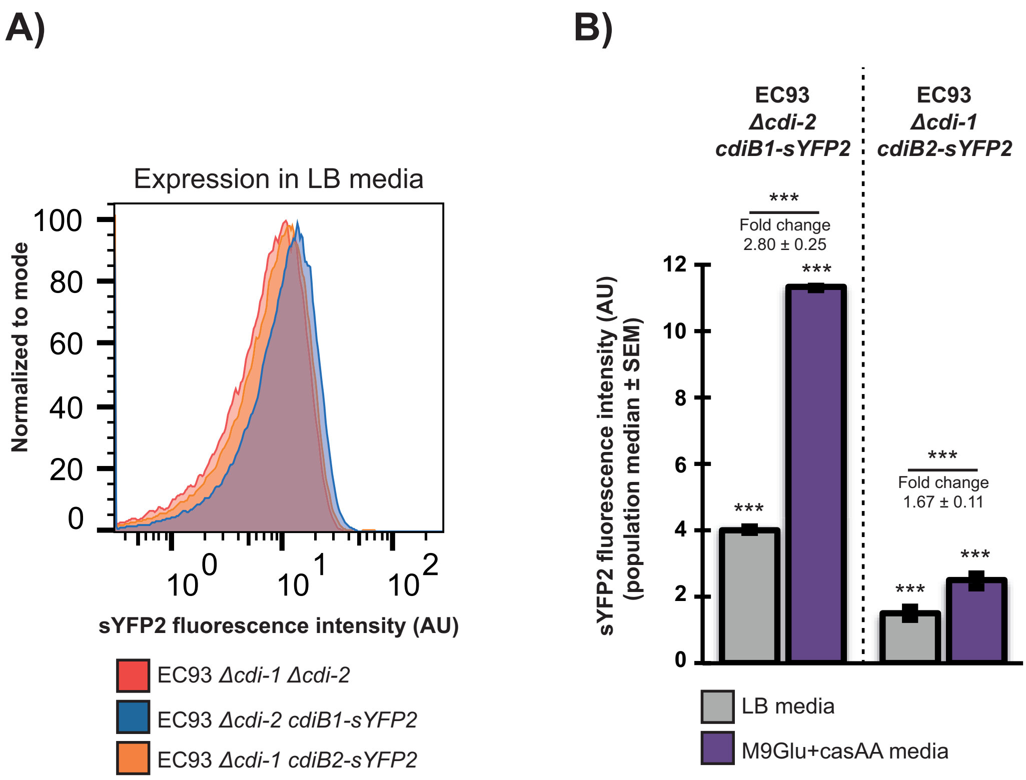

Figure S9. EC93 *cdiBAI1* and *cdiBAI2* expression in LB.

A) Relative transcriptional activity, measured as fluorescence of sYFP2 reporter inserted within the *cdi-1* or *cdi-2* loci in EC93 when cells are grown in LB media (n=3 biological replicates ±SEM). B) Quantification of data presented in panel A and a comparison of *cdi-1* and *cdi-2* expression between LB and M9Glu+casAA media (M9Glu+casAA expression data taken from Fig. 5BC). Statistical significance was determined by two-tailed, unpaired t-test (p-value: ***<0.0005).

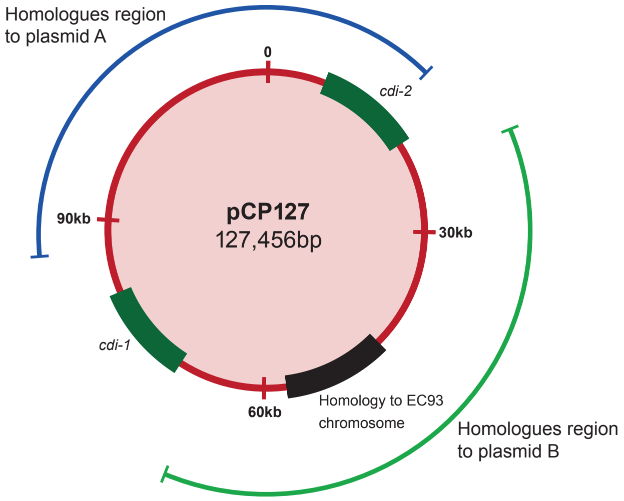

Figure S10. EC93 pCP127 plasmid homology

Schematic representation of the pCP127 plasmid of EC93 and the regions of homology to plasmid A and plasmid B from an *E. coli* isolate from pigs (GenBank accession no: CP010207.1), indicated by blue and green lines, respectively.

References

1. Arndt D, Grant JR, Marcu A, Sajed T, Pon A, Liang YJ, et al. PHASTER: a better, faster version of the PHAST phage search tool. Nucleic acids research. 2016;44(W1):W16-W21. doi: 10.1093/nar/gkw387. PubMed PMID: WOS:000379786800004.
2. Zhou Y, Liang YJ, Lynch KH, Dennis JJ, Wishart DS. PHAST: A Fast Phage Search Tool. Nucleic acids research. 2011;39:W347-W52. doi: 10.1093/nar/gkr485. PubMed PMID: WOS:000292325300056.
3. Willett JLE, Gucinski GC, Fatherree JP, Low DA, Hayes CS. Contact-dependent growth inhibition toxins exploit multiple independent cell-entry pathways. P Natl Acad Sci USA. 2015;112(36):11341-6. doi: 10.1073/pnas.1512124112. PubMed PMID: WOS:000360994900058.
4. Chiang SL, Rubin EJ. Construction of a mariner-based transposon for epitope-tagging and genomic targeting. Gene. 2002;296(1-2):179-85. doi: 10.1016/s0378-1119(02)00856-9. PubMed PMID: 12383515.
5. Datsenko KA, Wanner BL. One-step inactivation of chromosomal genes in Escherichia coli K-12 using PCR products. Proc Natl Acad Sci U S A. 2000;97(12):6640-5. doi: 10.1073/pnas.120163297. PubMed PMID: 10829079; PubMed Central PMCID: PMCPMC18686.
6. Datsenko KA, Wanner BL. One-step inactivation of chromosomal genes in Escherichia coli K-12 using PCR products. P Natl Acad Sci USA. 2000;97(12):6640-5. doi: DOI 10.1073/pnas.120163297. PubMed PMID: WOS:000087526300074.
7. Datta S, Costantino N, Court DL. A set of recombineering plasmids for gram-negative bacteria. Gene. 2006;379:109-15. doi: 10.1016/j.gene.2006.04.018. PubMed PMID: 16750601.
8. Virtanen P, Waneskog M, Koskiniemi S. Class II contact-dependent growth inhibition (CDI) systems allow for broad-range cross-species toxin delivery within the Enterobacteriaceae family. Mol Microbiol. 2019;111(4):1109-25. doi: 10.1111/mmi.14214. PubMed PMID: 30710431; PubMed Central PMCID: PMCPMC6850196.
9. Morse RP, Nikolakakis KC, Willett JLE, Gerrick E, Low DA, Hayes CS, et al. Structural basis of toxicity and immunity in contact-dependent growth inhibition

(CDI) systems. P Natl Acad Sci USA. 2012;109(52):21480-5. doi: 10.1073/pnas.1216238110. PubMed PMID: WOS:000313627700071.

10. Aoki SK, Pamma R, Hernday AD, Bickham JE, Braaten BA, Low DA. Contact-dependent inhibition of growth in Escherichia coli. Science. 2005;309(5738):1245-8. Epub 2005/08/20. doi: 10.1126/science.1115109. PubMed PMID: 16109881.

11. Ghosh A, Baltekin O, Waneskog M, Elkhalifa D, Hammarlof DL, Elf J, et al. Contact-dependent growth inhibition induces high levels of antibiotic-tolerant persister cells in clonal bacterial populations. EMBO J. 2018;37(9). doi: 10.15252/embj.201798026. PubMed PMID: 29572241; PubMed Central PMCID: PMCPMC5920241.

12. Ruhe ZC, Wallace AB, Low DA, Hayes CS. Receptor polymorphism restricts contact-dependent growth inhibition to members of the same species. MBio. 2013;4(4). doi: 10.1128/mBio.00480-13. PubMed PMID: 23882017; PubMed Central PMCID: PMC3735181.
